# Ruminant fat intake improves gut microbiota, serum inflammatory parameter and fatty acid profile in tissues of Wistar rats

**DOI:** 10.1038/s41598-021-98248-6

**Published:** 2021-09-23

**Authors:** Larissa de Brito Medeiros, Susana Paula Almeida Alves, Rui José Branquinho de Bessa, Juliana Késsia Barbosa Soares, Camila Neves Meireles Costa, Jailane de Souza Aquino, Gerlane Coelho Bernardo Guerra, Daline Fernandes de Souza Araújo, Lydiane Tavares Toscano, Alexandre Sérgio Silva, Adriano Francisco Alves, Mateus Lacerda Pereira Lemos, Wydemberg José de Araujo, Ariosvaldo Nunes de Medeiros, Celso José Bruno de Oliveira, Rita de Cassia Ramos do Egypto Queiroga

**Affiliations:** 1 Department of Nutrition, Federal University of Paraíba, João Pessoa, PB Brazil; 2 Centre for Interdisciplinary Research in Animal Health (CIISA), Faculty of Veterinary Medicine, University of Lisbon, Lisbon, Portugal; 3 Laboratory of Experimental Nutrition, Department of Nutrition, Federal University of Campina Grande, Cuité, CG Brazil; 4 Department of Biophysics and Pharmacology, Biosciences Centre, Federal University of Rio Grande Do Norte, Natal, Brazil; 5 Faculty of Health Sciences of Trairi, Federal University of Rio Grande Do Norte, Santa Cruz, Brazil; 6 Department of Physical Education, Health Sciences Centre, Federal University of Paraíba, João Pessoa, Brazil; 7 Department of Physiology and Pathology, Federal University of Paraíba, João PessoaParaíba, 58051-900 Brazil; 8 Department of Animal Science, Centre for Agrarian Sciences, Federal University of Paraíba, Areia, PB Brazil

**Keywords:** Cardiology, Health care, Risk factors

## Abstract

This study tested the hypothesis that naturally and industrially produced *trans*-fatty acids can exert distinct effects on metabolic parameters and on gut microbiota of rats. Wistar rats were randomized into three groups according to the diet: CONT-control, with 5% soybean oil and normal amount of fat; HVF-20% of hydrogenated vegetable fat (industrial); and RUM-20% of ruminant fat (natural). After 53 days of treatment, serum biochemical markers, fatty acid composition of liver, heart and adipose tissue, histology and hepatic oxidative parameters, as well as gut microbiota composition were evaluated. HVF diet intake reduced triglycerides (≈ 39.39%) and VLDL levels (≈ 39.49%). *Trans*-fatty acids levels in all tissue were higher in HVF group. However, RUM diet intake elevated amounts of anti-inflammatory cytokine IL-10 (≈ 14.7%) compared to CONT, but not to HVF. Furthermore, RUM intake led to higher concentrations of stearic acid and conjugated linoleic acid in all tissue; this particular diet was associated with a hepatoprotective effect. The microbial gut communities were significantly different among the groups. Our results show that ruminant fat reversed the hepatic steatosis normally caused by high fat diets, which may be related to the remodelling of the gut microbiota and its anti-inflammatory potential.

## Introduction

In recent decades, with the globalisation of food industries, there has been an increase in the availability of processed food products containing high amounts of hydrogenated vegetable fat (HVF), rich in industrially-produced *trans*-fatty acids (iTFA)^1^. *Trans*-fatty acids (TFA) are defined as unsaturated fatty acids with at least one unconjugated double bond with *trans* configuration^2^. In addition to being present in large quantities in processed products containing HVF, *trans-*fatty acids are also found naturally in small quantities in meat, milk and their derivatives^3–6^.

The negative effects on human health associated with iTFAs ingestion have been demonstrated since the 1990s by studies on cardiovascular risk indicators, such as increased LDL cholesterol, reduced HDL cholesterol, compromised endothelial function, and increased inflammation^7–9^. More recently, these negative effects of iTFAs consumption have been associated with imbalance in the gut microbiota leading to dysbiosis^10,11^.

On the other hand, ruminant TFA (rTFA) is naturally produced by the enzymes of microorganisms present in animals’ rumen through biohydrogenation, which is a complex process resulting in isomerisation, hydration or hydrogenation of unesterified unsaturated dietary FA^3^. After being produced, rTFA are absorbed and incorporated into body tissue and milk lipids^3,4^.

The biohydrogenation that occurs in the organism of ruminants produces various *trans* and *cis* isomeric FA, with *trans*-octadecenoates being quantitatively the most important group. Among the *trans*-octadecenoates from ruminant products, vaccenic acid (18:1*t*11) is usually the most abundant^6^. Conjugated linoleic acids (CLA), a group of geometric and positional conjugated isomers of linoleic acid, are also found in significant concentrations in ruminant animals’ milk and meat. Of the CLA, rumenic acid (18:2*c*9,*t*11), mostly derived from endogenous desaturation of 18:1*t*11, is the most commonly found CLA in ruminants^4^. Vaccenic acid (C18:1t11) and its rumenic acid derivative (CLA-c9t11) have shown positive effects on lipid and glucose metabolism, reflecting in lower cardiovascular risk^12,13^.

Although fats from industrial and natural sources contain the same *trans*- octadecenoates, the proportions are different. Elaidic acid (18:1*t*9) is the major isomer generated during industrial hydrogenation of oils; with the 18:1*t*6, 18:1*t*7, 18:1*t*8 and 18:1*t*10 isomers also predominant. This isomeric profile contrasts with that of rTFA, where often the 18:1*t*11 is the overwhelming *trans*-octadecenoate isomer^14^. For this reason, dietary fats may have distinct metabolic effects, according to the predominant FA types^15^. However, it is still unclear what is the impact of total ruminant fat intake and rTFA on the development of human chronic diseases related to gut microbiota^4^. There is still a lack of information if intake from different *trans*-fatty sources can modulate the gut microbiota and metabolic parameters, especially those related to the gut-liver axis. This axis refers to the bidirectional relationship between the intestine, including its microbiota, and the liver, which is the result of interactions between signals generated by dietary, genetic and environmental factors^16^.

In this context, health agencies from different countries and the World Health Organization (WHO), have joined forces to reduce TFA content in food and thus reduce its consumption^5,17,18^. In Brazil, regulations of the National Health Surveillance Agency (ANVISA) have resulted in a mandatory declaration of iTFA content to be placed on the labels on packaged foods, but the definition of *trans*-fat, comprising both iTFA and rTFA^19^, does not differentiate among the sources of these compounds. In parallel, research has been conducted on the manipulation of the profile of *trans*-octadecenoates in meat, to increase the concentration of vaccenic (C18:1t11) and rumenic (CLA-c9t11) acids^3,4^.

As changes in dietary FA composition can significantly affect several critical pathophysiological processes, related to the composition of the gut microbiota^20,21^, the present study aimed to test whether the intake of naturally (rTFA) and industrially-produced trans-fatty acids (iTFA) could have distinct effects on metabolic parameters and on the gut microbiota of rats.

## Materials and methods

### Animals and diets

Twenty-seven male Wistar rats (*Rattus norvegicus albinus*) were supplied by the Professor Thomas George Bioterium at the Institute for Research on Drugs and Medicines (IPeFarM) /Federal University of Paraíba (UFPB). The experiment was conducted at the Experimental Nutrition Lab (LANEX/CCS/UFPB) where the animals were kept in individual cages at 22 ± 2ºC with 12-h light:12-h dark cycles (lights on at 6:00 a.m.), with free access to water and fed ad libitum on a commercial diet (Presence-Purina®) until 60 days of age. Then rats, weighing between 200 and 350 g, were randomly assigned to the following three groups of 9 rats each: control diet (CONT), hydrogenated vegetable fat (HVF), and ruminant fat (RUM). Experimental diets were added with 20% hydrogenated vegetable fat or ruminant fat (ovine adipose tissues fat melted) into the control diet (Presence-Purina®). The RUM and HFV diets are considered high fat diet (HFD)^22^, while the CONT diet follows the nutritional recommendations for rodents^23^, with 5% soybean oil as a fat source. The proximal composition and energy value of the diets are shown in Table S1.

All animals were fed ad libitum throughout the 53-day experiment. The FA profile of the diets is presented in Table 1.

**Table 1:** Fatty acid (FA) content (mg/g dry matter) and profile (g/100 g of total FA) of control diet (CONT) and of diets with hydrogenated vegetable fat (HVF) or ruminant fat (RUM).

Fatty acids	Diets^1^		
	CONT	HVF	RUM
FA, mg/g dry matter	41	226	255
FA (g/100 g FA)			
**Saturated**			
10:0	–	0.07	0.10
12:0	–	1.08	0.06
i-14:0	–	–	0.05
14:0	0.10	0.58	2.35
i-15:0	–	–	0.24
a-15:0	–	–	0.21
15:0	–	–	0.39
i-16:0	–	–	0.15
16:0	14.1	13.4	21.7
i-17:0	–	–	0.29
a-17:0	–	–	0.48
17:0	0.12	0.12	0.99
18:0	3.18	9.24	29.8
20:0	0.37	0.35	0.21
22:0	0.31	0.40	0.07
23:0	–	0.05	–
24:0	–	0.17	–
**Monounsaturated**			
16:1c7	–	–	0.36
16:1c9	0.11	0.10	0.64
17:1c9	–	0.05	0.21
18:1t6/t7/t8	–	2.86	0.42
18:1t9	–	4.61	0.47
18:1t10	–	6.61	0.50
18:1t11	–	6.29	4.56
18:1t12	–	4.01	0.60
18:1c9	24.3	24.4	25.4
18:1c11	1.18	2.53	0.78
18:1c12	–	2.99	0.15
18:1c13	–	0.39	0.04
18:1t16	–	0.43	0.40
18:1c15	–	0.19	0.06
20:1c11	–	0.20	0.17
**Polyunsaturated**			
^2^18:2oi	–	1.85	0.29
18:2n-6	51.7	15.8	6.80
18:3n-3	4.5	1.30	0.69
18:2c9t11 (CLA)	–	–	0.50
^**3**^**Sums**			
BCFA	nd	nd	1.4
SFA	18.2	25.5	57.0
cis-MUFA	25.6	55.6	34.7
*trans-MUFA*	nd	24.8	7.5
PUFA	56.2	18.9	8.3

^1^CONT: Control; HVF: hydrogenated vegetable fat; RUM: ruminant fat.

^2^Sum of other isomers of 18:2n-6.

^3^BCFA: branched-chain fatty acids; SFA: saturated fatty acids; MUFA: monounsaturated fatty acids; PUFA: polyunsaturated fatty acids.

The hydrogenated vegetable fat was available commercially (Gordura vegetal Mesa®, S/A Fábrica de Produtos Alimentícios Vigor, São Paulo, Brazil) and the ruminant fat was melted from lamb adipose tissue of animals fed a diet designed to increase *trans* FA and conjugated linoleic acid at the Department of Animal Science, CCA, UFPB. All experimental methods were previously approved by The Commission on Animal Research and Ethics of the Center for Biotechnology of the University of Paraíba (CEUA-Cbiotec-UFPB n. 058/2017), in compliance with the regulations established by the National Council for the Control of Animal Experimentation (CONCEA, Brazil) by means of the Law No. 11.794/2008 (the Arouca Law), and with the Animal Research: Reporting of In Vivo Experiments (ARRIVE) guidelines 2.0 for in vivo animal experiments^24^.

### Body weight, feed intake, energy intake and energy efficiency

Animals were weighed weekly using a digital electronic scale (Toledo, prix III, São Bernardo do Campo, Brazil). Feed intake was evaluated three times per week and expressed as the difference between offered feed and residual feed. Based on the feed intake (g), the weekly energy intake was calculated according to the energy value offered by each diet. Energy efficiency was calculated as weight gained (final weight—initial weight) divided by energy intake (kcal).

### Euthanasia and tissue preparation for analysis

The animals were euthanized at 113 days of age, after being fed the experimental diet for 53 days. The animals had been fasted for 10 h and then weighed and anaesthetised with ketamine hydrochloride (75 mg) and xylazine hydrochloride (5 mg) administered via intraperitoneal injection. The rats were then euthanized by a section of the aorta arteries. Whole organs and tissues (heart, liver and adipose tissue) were removed, cleaned and weighed on an analytical balance. Organs and adipose tissue weights were normalized to 100 g of body weight. The organs and tissues of six animals from each group were kept at -80ºC until oxidative parameters, lipid profile and fatty acid analyses could be performed. The organs and tissues of the remaining three animals per group were washed in saline solution (0.9% NaCl) and fixed in 10% buffered formalin until histological analysis could be carried out.

### Blood serum biochemical parameters

Blood samples were collected after euthanasia from the animals and were left to rest for 30 min at room temperature (± 25 °C) before centrifugation^25^. Then blood samples were centrifuged (1,040 g for 15 min at 25 °C) to obtain serum samples that were kept at -80 ºC until biochemical analyses were performed. Total cholesterol and fraction of high density (HDL), low density (LDL) and very low-density lipoproteins (VLDL), triglycerides, glucose, alanine aminotransferase (ALT) and aspartate aminotransferase (AST) were quantified by commercial kits (Labtest, Minas Gerais, Brazil), according to manufacturer’s guidelines, using an automatic biochemical analyser LabMax 240 (Minas Gerais, Brazil).

The serum cytokine levels were evaluated using the protocols of the kits (R&D Systems-Haematology Group, Minneapolis, MN, USA), with standard capture and detection antibodies for IL-1β, TNF-α, and IL-10. The serum samples were homogenised with phosphate buffer (10 mM, pH 7.2–7.4), and centrifuged at 4000 g at 4 °C for 10 min so that the supernatant from the centrifugation could be used to determine the cytokines at an absorbance of 450 nm in a plate reader.

### Fatty acid in tissues and diets

Heart, liver and adipose tissue samples of 7 rats per treatment, randomly selected, were freeze-dried and sent to the Faculty of Veterinary Medicine at the University of Lisbon for FA analyses. Fatty acid methyl esters (FAME) and dimethyl acetals (DMA) from the tissue samples were prepared by reaction with HCl 1.25 M in methanol for 20 h at 50 °C. They were then analysed by gas chromatography with flame ionisation detection using a Shimadzu GC 2010-Plus (Shimadzu, Kyoto, Japan) equipped with a SP-2560 (100 m × 0.25 mm, 0.20 µm film thickness, Supelco, Bellefonte, PA, United States) capillary column. The chromatographic conditions were as follows: injector and detector temperatures were set at 250 and 280 °C, respectively; helium was used as the carrier gas at 1 mL/min constant flow; the initial oven temperature of 50 °C was held for 1 min, increased by 50 °C/min to 150 °C and held for 20 min; then increased by 1 °C/min to 190 °C; and finally increased by 2 °C/min to 220 °C and held for 40 min. Identification of FAME and DMA were achieved by comparison with commercial standards (FAME mix 37 components, Supelco Inc, Bellefont, PA, USA), by comparison with published chromatograms^26^ and by using electron impact mass spectrometry using a Shimadzu GC–MS QP2010 Plus (Shimadzu). The chromatographic column and the GC conditions used in the GC–MS were similar to the GC-FID analyses. Additional mass spectrometer conditions were as follows: ion source temperature, 200 °C; interface temperature, 240 °C; and emission voltage, 70 eV. The quantification of FA in the diets was performed as previously described. We provide short notation, systematic and common names in Table S2.

### Lipid peroxidation and antioxidant capacity in liver

Lipid peroxidation in liver was measured by the chromogenic product of 2-thiobarbituric acid (TBA) reaction with malondialdehyde (MDA), a product formed as a result of membrane lipid peroxidation^27^. The livers were homogenised with KCl (1:1), and samples of tissue homogenate (250 μL) were incubated at 37° C for 60 min. After that, the mixture was precipitated with 35% perchloric acid and centrifuged at 1207* g* for 20 min at 4 °C. Then, the supernatant was collected and 400 μL of 0.6% TBA was added and incubated at 95–100 °C for 1 h. After cooling, the samples were read in a spectrophotometer at a wavelength of 532 nm (Biospectro, SP-220 model-Brazil). MDA concentration was determined by substituting the absorbance values in the MDA standard curve obtained on the basis of a standard solution (1 μL of 1,1,3,3- tetramethoxypropane in 70 mL distilled water) diluted in series of 250, 500, 750, 1000, 1250, 1500, 1750, 2000, 2250, 2500, 2750, and 3000 μL of distilled water.

The liver homogenate was assembled as previously described. In addition, an aliquot of 1.25 mg of DPPH was diluted in ethanol (100 mL), kept under refrigeration, and protected from light. Then, 3.9 mL of DPPH solution was mixed with 100 μL of the supernatant liver homogenate in appropriate centrifuge tubes. These tubes were vortexed and left to stand for 30 min, then centrifuged at 1,207 g for 15 min at 20 °C. Then, the samples were read in a spectrophotometer at a wavelength of 515 nm (Biospectro, SP-220 model-Brazil)^28^. Results were expressed as the percentage of the oxidation inhibition: AOA = 100 − (((DPPH⋅ R) S/(DPPH⋅R) B) × 100), where (DPPH⋅R) S and (DPPH⋅R) B corresponding to the concentration of DPPH⋅ remaining after 30 min, measured in the sample (S) and blank (B) prepared with distilled water.

### Histological evaluation of liver

The livers of 3 rats per treatment were collected, washed in saline solution (0.9% NaCl) and fixed in 10% buffered formalin. The major lobe was subjected to a histological procedure according to the routine technique at the Pathology Laboratory (Department of Physiology and Pathology/CCS/UFPB) for obtaining blocks from which semi-serial 4-μm sections were cut. The slides were hydrated, stained with haematoxylin–eosin (HE), dehydrated, diaphanised in xylol and mounted with Entellan® for optical microscopic analysis (Motic BA 200, Olympus Optical Co, Philippines). Inflammatory exudate, hyperaemia, haemorrhage, necrosis, preservation of the hepatic parenchyma (cell integrity, centralised nuclei and highly evident nucleoli) and degenerative processes (e.g. fat degeneration) were evaluated in these liver histological Sects. ^29^.

For morphometric analysis, twenty random images from slides of liver tissues were used. Under an Axiolab light microscope (Zeiss) with 400 × resolution, twenty images were relayed to an image analysis system (Kontron Elektronik image analyser; Carl Zeiss, Germany—KS300 software). Reading of slides was performed randomly by two pathologists. A 10 × objective and 40 × photomicrograph of liver was used to obtain the images.

### Gut microbiota composition by high-throughput 16S rRNA sequencing

Individual stool samples from animals were collected on the three consecutive days just before euthanasia and immediately stored at − 20 °C. Three pooled samples from each treatment group were used for 16S rRNA amplicon sequencing. Genomic DNA was extracted using a commercial kit (PowerSoil DNA, Qiagen, Hilden, Germany). DNA integrity was assessed by electrophoresis in 1% agarose gel and quantified by fluorometry (Qubit, ThermoFisher, Waltham, MA, USA). The V3-V4 regions of the microbial 16S rRNA gene were amplified by PCR (95 °C for 3 min, followed by 25 cycles at 95 °C for 30 s, 55 °C for 30 s, 72 °C for 30 s and a final extension to 72 °C for 5 min) using the primers 341F: 5ʹ-TCG TCG GCA GCG TCA GAT GTG TAT AAG AGA CAG CCT ACG GGN GGC WGC AG-3ʹ and 785R: 5ʹ-GTC TCG TGG GCT CGG AGA TGT GTA TAA GAG ACA GGA CTA CHV GGG TAT CTA ATC C-3ʹ. Amplicon libraries were prepared using Nextera XT Index Kit (Illumina Inc., San Diego, CA, USA) and magnetic beads for cleaning and purification (Agencourt AMPureXP, Beckman Coulter, Indianapolis, USA). Paired-end sequencing was performed in Illumina MiSeq using a 500 cycle (2 × 250) V2 kit.

The raw demultiplexed paired-end sequences were downstream processed in QIIME 2 platform v.20.8^30^. Reads were filtered, normalized, denoised and parsed for non-chimeric sequences using DADA2^31^, producing Amplicon Sequence Variants (ASV) and an ASV respective feature table. For phylogeny, sequences were aligned using SEPP^32^ according to Greengenes database v.13.8. Alpha diversity was evaluated by Chao1, Simpson and Shannon diversity indices, while beta diversity was analysed by means of both weighted and unweighted Unifrac distance matrices. The visualisation plots of relative abundances, alpha and beta diversity metric measures were performed using phyloseq v.1.8.2^33^ in R v.3.5.7. Taxonomic classifications were attributed using the Naïve Bayes method based on Greengenes database v.13.8 with 99% of similarity for the V3-V4 regions^34^.

### Statistical analysis

Data from body weight, cumulative feed and energy intake of rats throughout the experiment were analysed using Proc MIXED from SAS 9.4. (SAS Institute Inc., Cary, NC, USA) applying random intercept and slope regression models to estimate the average daily gain (ADG), average daily feed intake (ADFI) and average daily energy intake (ADEI) (i.e., slopes of the respective random regression models). Repeated measurements with the same animal were modulated using an unstructured covariance matrix. Individual estimates of ADG, ADFI and ADEI were generated using the “estimate” statement and used to estimate feed or energy efficiency.

The efficiency, metabolic and FA variables were analysed with a simple linear model, implemented in the Proc MIXED, and considering the diet as the single fixed effect. The residuals for each treatment were checked for its normal distribution through QQ plots, Shapiro–Wilk test and Kolmogorov–Smirnov test using the Proc UNIVARIATE from SAS 9.4. For each variable that passes the normality check, the homogeneity of variances was evaluated using the Null Model Likelihood Ratio Test functionality of the Proc MIXED of SAS 9.4. When needed, the linear models accommodated heterogeneous variances patterns following the procedures described by Milliken & Johnson^35^. The pairwise multiple comparison of means was carried out using the Tukey adjustment method. The least square means, standard errors of the means, P and F values have been presented in all the tables.

Morphometric data of liver of hepatic steatosis (µm^2^) measured on the several slices from the same liver were averaged to obtain a mean value and then the analysis was conducted as described above but comparing only the treatments HVF and RUM, as CONT liver did not presented any lesions (mean = 0, variance = 0).

The variables that did not pass the normality test were analysed by non-parametric one way ANOVA on ranks, with the NPAR1WAY Procedure of SAS 9.4, where the Kruskal–Wallis Test, and pairwise multiple comparison were conducted using the Dwass. Steel, Critchlow-Fligner Method. The means, standard errors of the means, P and Chi square values have been placed in all the tables. Statistical significance was established at *P* < 0.05, with other ranges, that is, *P* < 0.01 and *P* < 0.001, *P* < 0.0001.

In terms of microbiota analyses, the non-parametric Kruskal–Wallis test was chosen for multiple comparisons of the alpha diversity indexes regarding richness (Chao1) and diversity (Shannon, Simpson). For beta diversity statistical analysis, the non-parametric Permutational multivariate analysis of variance (PERMANOVA) test was used to assess the dissimilarities of the microbial community structures across the experimental groups based on weighted (quantitative) and unweighted (qualitative) UniFrac distance matrices.

The differential abundance analysis was used to detect putative shifts in the abundance of specific Amplicon Sequence Variants (ASVs) across the experimental groups. It was performed by means of a machine learning classification method using the Random Forest with nested stratified k-fold cross-validation, providing automated hyperparameter optimisation, sample prediction and high feature classification accuracy. Taxonomic classification of each differential abundant feature was performed using BLASTn^36^ based on their respective amplicon sequence. All microbiota analyses were performed in QIIME 2 platform v.20.8.

## Results

### Body and tissues weights and feed intake, energy intake and energy efficiency

**Figure 1:**
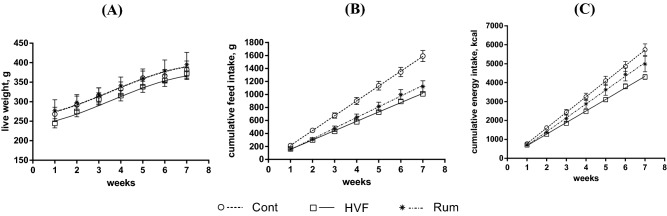
Body weight (BW) gain (**A**), cumulative feed (**B**) and energy intake (**C**) of adult rats fed CONT, HVF and RUM diets. Data were analysed using Proc MIXED from SAS 9.4. (SAS Institute Inc., Cary, NC, USA).

Body weight of rats and their cumulative feed and energy intake during the duration of the experiment are presented in Fig. 1. The slopes of the random regression models presented in Fig. 1 represent the average daily gain (ADG), feed (ADFI) and energy (ADEI) intakes are depicted in Table 2. The growth of rats was not affected by any of the diets, despite the differences observed for ADFI and ADEI. The rats fed HFD ingested less (*P* < 0.05) feed and energy than CONT fed rats. Nevertheless, the reduction in feed and energy intake was greater (*P* < 0.05) for HVF than for RUM fed rats. Thus, as ADG remained unaltered by diets, the differences observed in feed and energy intake are directly reflected in feed and energy efficiencies. Energy efficiency increased from 20 g LW/Mcal observed in CONT fed rats up to 23 g LW/Mcal for RUM and 28 g LW/Mcal for HVF fed rats. RUM and HFV rats ingested more lipids and less carbohydrates compared to CONT (Table S3).

**Table 2:** Body weight, feed and energy intake and conversion rate of rats fed CONT, HVF or RUM diets.

	Experimental Groups^1^			*P* value	F value
	CONT	HVF	RUM		
Initial bodyweight, g	268 ± 5.7	245 ± 11.7	277 ± 8.8	–	–
Final bodyweight, g	374 ± 4.9	367 ± 17.2	389 ± 10.2	0.378	1.01*
^2^ADG, g/d	2.30 ± 0.142	2.54 ± 0.157	2.47 ± 0.149	0.486	0.73§
^3^ADFI, g/d	32.6^a^ ± 0.60	20.5^c^ ± 0.63	23.5^b^ ± 0.60	< 0.001	101§
^4^ADEI, kcal/d	118^a^ ± 2.48	87^c^ ± 2.61	104^b^ ± 2.48	< 0.001	36.5§
Feed efficiency, g/kg	73^c^ ± 0.14	120^a^ ± 0.58	99^b^ ± 0.32	< 0.001	55.0*
Energy efficiency, g/Mcal	20^c^ ± 0.04	28^a^ ± 0.14	23^b^ ± 0.07	< 0.001	18.6*

Averages on the same line with significant differences (*P* < 0.05) are indicated by different letters.

^1^CONT: Control; HVF: hydrogenated vegetable fat; RUM: ruminant fat.

^2^ADG: average daily gain; ADFI: average daily feed intake; ADEI: average daily energy intake.

*F_(2,26)_ values; §F_(2,184)_.

The weight of the hearts and livers did not differ among treatments, while the HVF presented lower accumulation of adipose tissue (*P* < 0.05) than the CONT, but not to RUM (Table S4).

### Blood serum biochemical parameters

There were no significant differences regarding glucose, total cholesterol, HDL, LDL, ALT and AST serum parameters among the dietary treatments. However, HVF presented lower serum triglycerides and VLDL than CONT and RUM (*P* < 0.05) (Table 3).

**Table 3:** Serum biochemical parameters and hepatic oxidative parameters of rats fed CONT, HVF or RUM diets.

	Experimental Groups^1^			*P* value	F_(2,26)_ value
	CONT	HVF	RUM		
**Parameters (mg/dL)**					
Glucose	229 ± 15.8	214 ± 16.7	216 ± 15.8	0.771	0.26
Triglycerides	49.0^a^ ± 4.39	29.7^b^ ± 4.62	61.6^a^ ± 12.1	0.007	6.11
Total cholesterol	43.2 ± 3.86	40.4 ± 4.07	43.7 ± 1.86	0.769	0.26
^**2**^**Lipoproteins (mg/dL)**					
HDL	34.1 ± 2.48	33.1 ± 2.62	35.1 ± 2.48	0.859	0.15
LDL	8.2 ± 1.08	6.3 ± 1.20	6.6 ± 1.08	0.447	0.87
VLDL	9.8^a^ ± 1.09	5.9^b^ ± 0.59	12.3^a^ ± 2.41	0.003	7.40
^**3**^**Transaminases (U/L)**					
ALT	47.8 ± 2.69	52.1 ± 2.83	52.0 ± 2.69	0.452	0.82
AST	102 ± 9.0	130 ± 9.5	136 ± 18.1	0.077	2.84
^**4**^**Cytokines (pg/mL)**					
IL-10	0.68^b^ ± 0.032	0.72^ab^ ± 0.033	0.78^a^ ± 0.017	0.024	4.32
IL-ß	0.25 ± 0.026	0.24 ± 0.028	0.26 ± 0.026	0.862	0.89
TNF-α	0.59 ± 0.014	0.62 ± 0.014	0.59 ± 0.014	0.423	0.15
^**5**^**Hepatic oxidative parameters**					
MDA in liver (µmol/g tissue)	1.03 ± 0.054	1.08 ± 0.065	0.93 ± 0.054	0.222	1.65*
CAT in liver (%)	91.6 ± 0.88	92.6 ± 2.79	90.0 ± 2.58	0.764	0.27*

^1^CONT: Control; HVF: hydrogenated vegetable fat; RUM: ruminant fat.

^2^HDL: high density lipoprotein; LDL: low density lipoprotein; VLDL: very low density lipoprotein.

^3^ALT: alanine aminotransferase; AST: aspartate aminotransferase. Averages on the same line with significant differences (*P* < 0.05) are indicated by different letters.

^4^IL-10: Interleukin 10; IL-1β: Interleukin 1β; TNF-α: Alpha Tumour Necrosis Factor.

^5^MDA: malondialdehyde; CAT: total antioxidant capacity.

*F_(2,17)_ values.

In relation to serum cytokine levels, there were no significant differences regarding IL-1β and TNF-α among the dietary treatments. Nevertheless, it was observed that RUM presented higher amounts of IL-10 compared to CONT, but not HVF (*P* < 0.05) (Table 3).

### Fatty acid in tissue

The detailed FA composition (mg/g dry tissue) of the liver, adipose tissues and heart of rats are presented in Tables 4, 5, and 6, respectively. As expected, the diets had a profound effect on FA composition in all tissues studied. Some groups of FA showed a similar pattern of response to treatments across tissues. Thus, in all tissues, the total *trans*-MUFA was highest (*P* < 0.05) for HVF treatment than the for other treatments. The amount of 18:1*t*11 in the heart was similar in HVF and RUM treatments and lower in the CONT. Tissues from rats from HVF treatment also presented higher concentration of some *cis*-octadecenoic isomer like 18:1c12 and 18:1c13 as well as the sum of 18:2 isomers (18:2oi, most of them with at least one *trans* double bond). In all tissues, the RUM treatment resulted in the highest concentration of 18:0, 18:2c9t11 (CLA), and most of the branched-chain FA (BCFA), different from the other treatments. The 18:1c9 was also highest with RUM than with the other treatments in heart and adipose tissue but not in the liver.

**Table 4:** Fatty acid composition (mg/g dry tissue) of liver of adult rats fed CONT, HVF or RUM diets.

Fatty acids	Experimental Groups^1^			*P* value	F_(2,17)_ value/χ^2^
	CONT	HVF	RUM		
14:0	0.41 ± 0.257	0.12 ± 0.020	0.18 ± 0.039	0.408	1.79
15:0	0.16^a^ ± 0.036	0.07^b^ ± 0.007	0.15^a^ ± 0.022	0.003	12.0
i-16:0	0.04^ab^ ± 0.01	0.01^b^ ± 0.01	0.05^a^ ± 0.01	0.007	9.95
16:0	20.1^a^ ± 4.52	10.3^b^ ± 0.86	12.7^ab^ ± 1.05	0.008	9.55
16:1-*t*	0.02^c^ ± 0.006	0.42^a^ ± 0.054	0.16^b^ ± 0.031	< 0.001	16.54
i-17:0	0.05 ± 0.013	0.06 ± 0.081	0.08 ± 0.063	0.036	6.64
16:1-*c*7	0.13 ± 0.041	0.24 ± 0.015	0.18 ± 0.025	0.026	7.29
16:1-*c*9	1.37^a^ ± 0.765	0.13^b^ ± 0.030	0.19^b^ ± 0.026	0.003	11.86
a-17:0	0.04^b^ ± 0.011	nd	0.11a ± 0.015	< 0.001	16.19
17:0	0.45^a^ ± 0.019	0.17^b^ ± 0.013	0.50^a^ ± 0.033	0.002	12.45
i-18:0	0.23^a^ ± 0.034	0.05^b^ ± 0.007	0.04^b^ ± 0.007	< 0.001	14.56
17:1-*c*9	0.07^a^ ± 0.023	0.01^b^ ± 0.008	0.06^a^ ± 0.009	0.013	8.76
18:0	23.1^b^ ± 0.08	19.8^b^ ± 1.59	27.7^a^ ± 0.41	< 0.001	21.68
18:1-*t*6/-*t*7/-*t*8	0.03^c^ ± 0.028	0.63^a^ ± 0.080	0.11^b^ ± 0.012	< 0.001	48.13
18:1-*t*9	0.08^c^ ± 0.016	1.73^a^ ± 0.165	0.17^b^ ± 0.014	< 0.001	15.77
18:1-*t*10	0.04^b^ ± 0.012	0.96^a^ ± 0.117	0.10^b^ ± 0.018	< 0.001	14.79
18:1-*t*11	0.18^c^ ± 0.006	1.83^a^ ± 0.164	1.05^b^ ± 0.108	< 0.001	82.76
18:1-*t*12	0.06^c^ ± 0.008	2.39^a^ ± 0.163	0.30^b^ ± 0.024	< 0.001	142.7
18:1-*c*9	12.7 ± 6.23	8.4 ± 1.18	10.5 ± 1.91	0.608	0.99
18:1-*t*15	nd	0.18^a^ ± 0.051	0.10^b^ ± 0.010	0.032	4.59
18:1-*c*11	2.17^a^ ± 0.321	1.38^b^ ± 0.103	1.01^c^ ± 0.038	< 0.001	15.44
18:1-*c*12	0.13^b^ ± 0.017	0.93^a^ ± 0.082	0.07^c^ ± 0.009	< 0.001	57.53
18:1-*c*13	0.05^b^ ± 0.010	0.12^a^ ± 0.011	0.04^b^ ± 0.010	< 0.001	17.57
18:1-*t*16/-*c*14	0.04^b^ ± 0.005	0.16^a^ ± 0.015	0.18^a^ ± 0.014	0.001	13.27
18:1-*c*15	nd	0.05a ± 0.004	0.02b ± 0.004	< 0.001	27.8
^2^18:2oi	0.03^c^ ± 0.009	0.39^a^ ± 0.059	0.11^b^ ± 0.016	< 0.001	25.38
18:2n-6	18.8 ± 3.71	14.1 ± 1.07	12.3 ± 0.99	0.179	1.90
20:0	0.09 ± 0.024	0.05 ± 0.006	0.05 ± 0.006	0.246	1.53
18:3n-6	0.13 ± 0.023	0.08 ± 0.012	0.12 ± 0.011	0.065	3.23
20:1	0.18^a^ ± 0.046	0.06^b^ ± 0.009	0.05^b^ ± 0.004	0.002	12.58
18:3n-3/20:1-*c*11	0.41 ± 0.169	0.18 ± 0.024	0.20 ± 0.038	0.129	4.10
18:2-*c*9*t*11	0.03^b^ ± 0.015	0.06^b^ ± 0.015	0.16^a^ ± 0.036	0.004	11.0
20:2n-6	0.23^a^ ± 0.020	0.14^b^ ± 0.010	0.12^b^ ± 0.020	0.002	9.12
20:3n-9	0.07^b^ ± 0.014	0.08^b^ ± 0.007	0.35^a^ ± 0.029	0.001	13.81
22:0	0.07 ^b^ ± 0.012	0.12^a^ ± 0.015	0.13^ab^ ± 0.024	0.023	7.49
20:3n-6	0.41^b^ ± 0.027	0.75^ab^ ± 0.096	0.94^a^ ± 0.138	< 0.001	12.44
20:4n-6	23.9 ± 1.68	23.3 ± 1.77	25.3 ± 1.02	0.797	0.454
23:0	0.16 ± 0.009	0.14 ± 0.026	0.13 ± 0.020	0.338	2.17
20:5n-3	0.08^b^ ± 0.009	0.09^ab^ ± 0.032	0.19^a^ ± 0.030	0.008	6.58
22:4n-6	0.35^a^ ± 0.033	0.22^b^ ± 0.035	0.26^ab^ ± 0.033	0.040	3.92
22:5n-6	0.19^a^ ± 0.033	0.06^b^ ± 0.018	0.13^a^ ± 0.017	0.008	6.55
22:5n-3	0.41 ± 0.034	0.45 ± 0.082	0.47 ± 0.076	0.719	0.34
22:6n-3	3.48 ± 0.300	2.81 ± 0.324	3.04 ± 0.300	0.320	1.22
^3^Sums					
BCFA	0.36^ab^ ± 0.066	0.12^b^ ± 0.015	0.28^a^ ± 0.025	0.003	11.8
SFA	44.9^a^ ± 5.43	31.0^b^ ± 2.43	41.8^a^ ± 1.37	0.009	9.38
*cis*-MUFA	16.6 ± 7.39	11.3 ± 1.39	12.1 ± 2.01	0.923	0.16
*trans*-MUFA	0.41^c^ ± 0.035	8.15^a^ ± 0.734	1.99^b^ ± 0.205	< 0.001	16.9
n-6 PUFA	44.0 ± 2.40	38.7 ± 2.59	39.2 ± 2.40	0.265	1.44
n-3 PUFA	4.38 ± 0.314	3.53 ± 0.339	3.90 ± 0.314	0.206	1.74
PUFA	48.5 ± 2.50	42.7 ± 2.70	43.7 ± 2.50	0.260	1.46

Averages on the same line with significant differences (*P* < 0.05) are indicated by different letters.

^1^CONT: Control; HVF: hydrogenated vegetable fat; RUM: ruminant fat.

^2^Sum of other isomers of 18:2n-6.

^3^BCFA: branched-chain fatty acids; SFA: saturated fatty acids; MUFA: monounsaturated fatty acids; PUFA: polyunsaturated fatty acids.

^†^Variables analysed with non-parametric Kruskal–Wallis Test and is the χ^2^ that is presented.

**Table 5:** Fatty acid composition of the adipose tissue of rats fed CONT, HVF or RUM diets (mg/g dry matter).

Fatty acids	Experimental Groups^1^			*P* value	F_(2,17)_ value/χ^2^
	CONT	HVF	RUM		
12:0	0.60^b^ ± 0.050	3.91^a^ ± 0.207	0.59^b^ ± 0.027	< 0.001	126.6
i-14:0	0.12 ± 0.023	nd	0.43 ± 0.023	< 0.001	91.44
14:0†	10.5^b^ ± 0.62	5.30^c^ ± 0.369	13.0^a^ ± 0.286	< 0.001	14.79
i-15:0	0.29 ± 0.046	nd	1.01 ± 0.046	< 0.001	120.3
a-15:0†	0.16 ± 0.025	nd	1.06 ± 0.063	0.003	9.00
14:1-*c*9†	0.72^a^ ± 0.081	0.21^c^ ± 0.016	0.40^b^ ± 0.059	< 0.001	13.55
15:0	2.59^b^ ± 0.367	1.04^c^ ± 0.065	4.11^a^ ± 0.0	< 0.001	595.2
i-16:0	0.82^b^ ± 0.041	0.24^c^ ± 0.027	1.54^a^ ± 0.041	< 0.001	361.4
16:0	231^a^ ± 11.6	118^c^ ± 6.8	180^b^ ± 2.4	< 0.001	44.38
16:1-*t*	0.43^c^ ± 0.067	3.92^a^ ± 0.157	2.06^b^ ± 0.062	< 0.001	286.6
i-17:0	0.71^b^ ± 0.069	0.53^b^ ± 0.074	2.17^a^ ± 0.069	< 0.001	165.3
16:1-*c*7†	3.64^a^ ± 0.295	2.09^b^ ± 0.159	4.53^a^ ± 0.124	< 0.001	14.22
16:1-*c*9	37.5^a^ ± 2.13	10.1^b^ ± 2.30	17.9^b^ ± 2.13	< 0.001	41.67
a-17:0	0.68^b^ ± 0.049	0.21^b^ ± 0.016	2.74^a^ ± 0.145	< 0.001	186.5
17:0	2.47^b^ ± 0.280	1.44^b^ ± 0.056	6.08^a^ ± 0.280	< 0.001	136.7
i-18:0	1.04^a^ ± 0.091	0.36^b^ ± 0.098	0.78^a^ ± 0.091	0.001	12.95
17:1-*c*9	1.96^a^ ± 0.152	0.83^b^ ± 0.094	3.11^a^ ± 0.152	< 0.001	85.52
18:0	37.2^b^ ± 2.33	43.0^b^ ± 2.74	110^a^ ± 9.55	< 0.001	27.54
18:1-*t*6/-*t*7/-*t*8	0.25^c^ ± 0.021	20.0^a^ ± 0.74	3.10^b^ ± 0.110	< 0.001	675.7
18:1-*t*9	0.53^c^ ± 0.035	33.3^a^ ± 1.02	4.02^b^ ± 0.169	< 0.001	709.9
18:1-*t*10†	0.54^c^ ± 0.085	27.8^a^ ± 0.72	3.57^b^ ± 0.891	< 0.001	16.90
18:1-*t*11	1.6^c^ ± 0.14	33.6^a^ ± 1.79	26.9^b^ ± 1.65	< 0.001	274.6
18:1-*t*12	0.66^c^ ± 0.193	26.5^a^ ± 0.48	2.39^b^ ± 0.193	< 0.001	1293.4
18:1-*c*9	330^b^ ± 15.1	281^c^ ± 6.9	432^a^ ± 4.4	0.003	274.4
18:1-*t*15	nd	8.51 ± 0.544	1.88 ± 0.220	0.004	8.30
18:1-*c*11	24.1^a^ ± 1.46	24.2^a^ ± 0.49	14.9^b^ ± 0.45	< 0.001	103.0
18:1-*c*12	0.52^c^ ± 0.089	20.1^a^ ± 0.206	1.16^b^ ± 0.091	< 0.001	4013
18:1-*c*13†	0.67^b^ ± 0.052	2.36^a^ ± 0.070	0.32^c^ ± 0.071	< 0.001	16.90
18:1-*t*16/-*c*14	0.15^b^ ± 0.018	1.43^a^ ± 0.112	1.68^a^ ± 0.104	< 0.001	164.0
18:1-*c*15	nd	0.73^a^ ± 0.039	0.19^b^ ± 0.014	< 0.001	171.0
^2^18:2oi	0.87^c^ ± 0.122	13.3^a^ ± 0.287	3.57^b^ ± 0.122	< 0.001	811.1
18:2n-6	301^a^ ± 15.6	186^b^ ± 3.2	146^c^ ± 2.9	< 0.001	80.03
19:1	0.25^c^ ± 0.033	0.91^b^ ± 0.037	1.36^a^ ± 0.051	< 0.001	193.1
20:0	0.80 ± 0.102	0.88 ± 0.109	0.90 ± 0.109	0.772	0.26
18:3n-6	0.79^a^ ± 0.040	0.23^b^ ± 0.043	0.28^b^ ± 0.040	< 0.001	58.88
18:3n-3/20:1-*c*11	14.6^a^ ± 0.69	8.99^b^ ± 0.532	7.92^c^ ± 0.493	< 0.001	52.74
20:1	2.55^a^ ± 0.363	1.07^b^ ± 0.181	1.45^b^ ± 0.166	0.012	6.70
18:2-*c*9*t*11	0.95^c^ ± 0.073	4.23^b^ ± 0.465	9.26^a^ ± 0.309	< 0.001	361.9
20:2n-6	1.60^a^ ± 0.163	0.35^b^ ± 0.035	0.36^b^ ± 0.044	< 0.001	28.46
20:3n-9	0.36^a^ ± 0.025	0.08^b^ ± 0.035	0.36^a^ ± 0.033	< 0.001	22.80
22:0	0.19^b^ ± 0.032	0.36^a^ ± 0.032	0.15^b^ ± 0.044	< 0.001	12.07
20:3n-6	1.00^a^ ± 0.102	0.25^b^ ± 0.030	0.31^b^ ± 0.028	< 0.001	24.74
20:4n-6	5.54^a^ ± 0.515	1.26^b^ ± 0.109	1.55^b^ ± 0.101	< 0.001	33.36
20:5n-3	0.23^a^ ± 0.033	0.06^b^ ± 0.008	0.05^b^ ± 0.012	0.001	12.88
24:0	0.17 ± 0.020	0.16 ± 0.020	0.11 ± 0.024	0.116	2.52
22:4n-6	1.33^a^ ± 0.185	0.14^b^ ± 0.028	0.20^b^ ± 0.026	< 0.001	20.41
22:5n-6	0.53 ± 0.062	nd	nd	–	
22:5n-3	0.84^a^ ± 0.079	0.16^b^ ± 0.039	0.23^b^ ± 0.32	< 0.001	30.91
22:6n-3	1.49^a^ ± 0.144	0.21^b^ ± 0.050	0.24^b^ ± 0.045	< 0.001	36.64
^**3**^**Sums**					
BCFA	3.79^b^ ± 0.212	1.35^c^ ± 0.101	9.74^a^ ± 0.417	< 0.001	227.0
SFA	290^a^ ± 14.5	180^b^ ± 8.9	324^a^ ± 14.5	< 0.001	44.87
*cis*-MUFA	399^b^ ± 18.4	342^c^ ± 5.8	474^a^ ± 5.4	< 0.001	142.0
*trans*-MUFA	4.0^c^ ± 0.26	154^a^ ± 5.2	45.3^b^ ± 2.15	< 0.001	593.2
n-6 PUFA	312^a^ ± 16.5	188^b^ ± 3.2	148^c^ ± 2.9	< 0.001	79.92
n-3 PUFA	17.2^a^ ± 0.89	9.32^b^ ± 0.416	8.33^b^ ± 0.385	< 0.001	42.00
PUFA†	332^a^ ± 17.4	216^b^ ± 4.6	170^c^ ± 2.7	< 0.001	16.90

Averages on the same line with significant differences (*P* < 0.05) are indicated by different letters.

^1^CONT: Control; HVF: hydrogenated vegetable fat; RUM: ruminant fat.

^2^Sum of other isomers of 18:2n-6.

^3^BCFA: branched-chain fatty acids; SFA: saturated fatty acids; MUFA: monounsaturated fatty acids; PUFA: polyunsaturated fatty acids.

^†^Variables analysed with non-parametric Kruskal–Wallis Test and is the χ^2^ that is presented.

**Table 6:** Fatty acid composition of the heart tissue of rats fed CONT, HVF or RUM diets (mg/g dry matter).

Fatty acids	Experimental Groups^1^			*P* value	F_(2,16)_ value/χ^2^
	CONT	HVF	RUM		
14:0	0.22^b^ ± 0.071	0.28^b^ ± 0.084	1.11^a^ ± 0.169	< 0.001	11.97
i-15:0	nd	nd	0.09 ± 0.008	–	–
a-15:0	nd	nd	0.10 ± 0.011	–	–
14:1-*c*9†	0.016 ± 0.0061	nd	0.029 ± 0.0056	0.179	1.80
15:0	0.07^b^ ± 0.008	0.05^b^ ± 0.009	0.28^a^ ± 0.028	< 0.001	17.68
i-16:0	0.03^b^ ± 0.005	0.01^b^ ± 0.006	0.12^a^ ± 0.016	< 0.001	21.86
16:0	9.57^b^ ± 1.312	9.87^b^ ± 1.55	16.6^a^ ± 1.31	0.003	8.72
16:1-*t*	nd	0.25 ± 0.029	nd	–	–
i-17:0†	0.03^b^ ± 0.003	0.02^b^ ± 0.003	0.16^a^ ± 0.022	< 0.002	13.05
16:1-*c*7	0.07^b^ ± 0.008	0.09^b^ ± 0.019	0.32^a^ ± 0.047	< 0.001	13.83
16:1-*c*9†	0.53^ab^ ± 0.180	0.16^b^ ± 0.098	0.64^a^ ± 0.059	0.010	9.23
a-17:0†	0.02^b^ ± 0.003	0.01^c^ ± 0.004	0.26^a^ ± 0.034	< 0.001	14.87
17:0	0.25^b^ ± 0.023	0.12^c^ ± 0.028	0.64^a^ ± 0.069	< 0.001	25.99
i-18:0	0.09 ± 0.017	0.09 ± 0.020	0.09 ± 0.017	0.990	0.01
17:1-*c*9†	0.04^b^ ± 0.074	0.08^ab^ ± 0.030	0.16^a^ ± 0.025	0.010	9.27
18:0	16.8^b^ ± 0.69	15.7^b^ ± 0.82	27.7^a^ ± 1.54	< 0.001	24.66
18:1-*t*6/-*t*7/-*t*8†	0.15^b^ ± 0.150	0.94^a^ ± 0.178	0.22^b^ ± 0.028	0.004	11.08
18:1-*t*9†	0.37^b^ ± 0.322	2.52^a^ ± 0.381	0.38^b^ ± 0.054	0.003	11.79
18:1-*t*10†	0.20^b^ ± 0.191	1.37^a^ ± 0.328	0.29^b^ ± 0.106	0.006	10.32
18:1-*t*11†	0.41^b^ ± 0.293	2.17^a^ ± 0.315	2.44^a^ ± 0.264	0.011	8.97
18:1-*t*12†	0.31^b^ ± 0.226	1.88^a^ ± 0.268	0.32^b^ ± 0.009	0.004	11.09
18:1-*c*9	7.47^b^ ± 1.973	12.4^b^ ± 2.335	29.7^a^ ± 4.132	< 0.001	11.85
18:1-*t*15†	0.09^b^ ± 0.086	0.51^a^ ± 0.102	0.17^b^ ± 0.026	0.006	10.32
18:1-*c*11	2.27 ± 0.110	2.37 ± 0.130	2.11 ± 0.110	0.333	1.18
18:1-*c*12†	0.23^b^ ± 0.167	1.27^a^ ± 0.198	0.11^b^ ± 0.014	0.004	11.08
18:1-*c*13†	0.05^b^ ± 0.019	0.18^a^ ± 0.003	0.03^b^ ± 0.023	0.009	9.51
18:1-*t*16/-*c*14†	0.03^b^ ± 0.014	0.15^a^ ± 0.02	0.17^a^ ± 0.014	0.002	12.90
18:1-*c*15†	0.01^b^ ± 0.005	0.05^a^ ± 0.003	0.02^b^ ± 0.003	0.001	13.26
18:2n-6	18.1 ± 1.16	19.0 ± 1.37	18.2 ± 1.16	0.859	0.15
20:0	0.07^b^ ± 0.005	0.10^ab^ ± 0.020	0.12^a^ ± 0.017	0.011	6.14
18:3n-6†	0.02 ± 0.003	0.01 ± 0.004	0.02 ± 0.003	0.125	4.15
18:3n-3/20:1-*c*11	0.24^b^ ± 0.057	0.33^ab^ ± 0.068	0.51^a^ ± 0.057	0.014	5.61
18:2-*c*9*t*11	0.02^b^ ± 0.007	0.06^b^ ± 0.026	0.43^a^ ± 0.056	< 0.001	27.68
20:2n-6	0.14^a^ ± 0.016	0.07^b^ ± 0.008	0.09^b^ ± 0.006	0.006	7.17
20:3n-9	0.02^b^ ± 0.003	0.01^b^ ± 0.004	0.09^a^ ± 0.003	< 0.001	153.53
22:0†	0.03^b^ ± 0.008	0.07^a^ ± 0.009	0.05^ab^ ± 0.008	0.117	4.28
20:3n-6	0.22^b^ ± 0.023	0.32^a^ ± 0.027	0.38^a^ ± 0.023	< 0.001	12.78
20:4n-6	15.9 ± 0.99	14.4 ± 0.26	15.3 ± 0.99	0.288	1.35
20:5n-3	0.028^ab^ ± 0.0027	0.023^b^ ± 0.0033	0.042^a^ ± 0.0059	0.038	4.01
24:0	0.033^ab^ ± 0.0033	0.045^b^ ± 0.0053	0.032^a^ ± 0.0033	0.111	2.53
22:4n-6	0.69 ± 0.035	0.64 ± 0.042	0.64 ± 0.035	0.586	0.55
22:5n-6	0.58 ± 0.064	0.41 ± 0.076	0.50 ± 0.064	0.270	1.42
22:5n-3†	1.01^b^ ± 0.078	1.67^a^ ± 0.207	1.74^a^ ± 0.174	0.004	10.89
22:6n-3	6.67^b^ ± 0.490	9.08^a^ ± 0.580	8.11^ab^ ± 0.490	0.017	5.28
^**2**^**Sums**					
BCFA	0.17^b^ ± 0.025	0.13^b^ ± 0.031	0.81^a^ ± 0.093	< 0.001	24.16
SFA†	27.2^b^ ± 1.64	26.4^b^ ± 1.94	47.3^a^ ± 3.53	< 0.001	12.73
*cis*-MUFA	10.6^b^ ± 2.18	15.5^b^ ± 2.57	33.2^a^ ± 4.39	0.001	10.66
*trans*-MUFA†	1.56^b^ ± 1.320	9.63^a^ ± 1.580	3.83^b^ ± 0.459	0.004	11.09
n-6 PUFA	35.6 ± 1.22	34.9 ± 1.44	35.1 ± 1.22	0.915	0.09
n-3 PUFA	8.0^b^ ± 0.25	11.1^a^ ± 0.78	10.4^a^ ± 0.66	< 0.001	12.22
PUFA	43.6 ± 1.43	46.1 ± 1.69	46.0 ± 1.43	0.426	0.90

Averages on the same line with significant differences (*P* < 0.05) are indicated by different letters.

^1^CONT: Control; HVF: hydrogenated vegetable fat; RUM: ruminant fat.

^2^BCFA: branched-chain fatty acids; SFA: saturated fatty acids; MUFA: monounsaturated fatty acids; PUFA: polyunsaturated fatty acids.

^†^Variables analysed with non-parametric Kruskal–Wallis Test and is the χ^n2^ that is presented.

In the liver, and excluding the 18:0 and the 16:0, all the other main FA (i.e. 18:1c9, 18:2n-6, 20:4n-6, and 22:6n-3) did not differ among treatments. In the adipose tissue, the HVF group presented the lowest concentration of 16:0, and of total SFA, whereas both HVF and RUM presented lower concentrations of most PUFA, including 18:2n-6, 20:4n-6, 20:5n-3 and 22:6n-3 than CONT. The concentration of total FA in the liver averaged 101 ± 11.1 mg/g dry tissue and did not differ among treatments.

In the heart, the RUM treatment resulted in a general larger (*P* < 0.001) deposit of FA than in the other treatments; these were similar (131 ± 6.7, 99 ± 7.9 and 83 ± 6.7 mg/g dry tissue, respectively for RUM, HVF and CONT). As a consequence, RUM treatment presented the highest concentration of SFA and *cis*-MUFA, although similar n-6 PUFA, when compared to the other treatments. The content of n-3 PUFA was higher in both HVF and RUM and lower in CONT.

### Assessment of lipid peroxidation levels and antioxidant capacity assay in liver

MDA content and antioxidant capacity in liver did not differ among treatments (*P* > 0.05) (Table 3).

### Histological evaluation of liver

**Figure 2:**
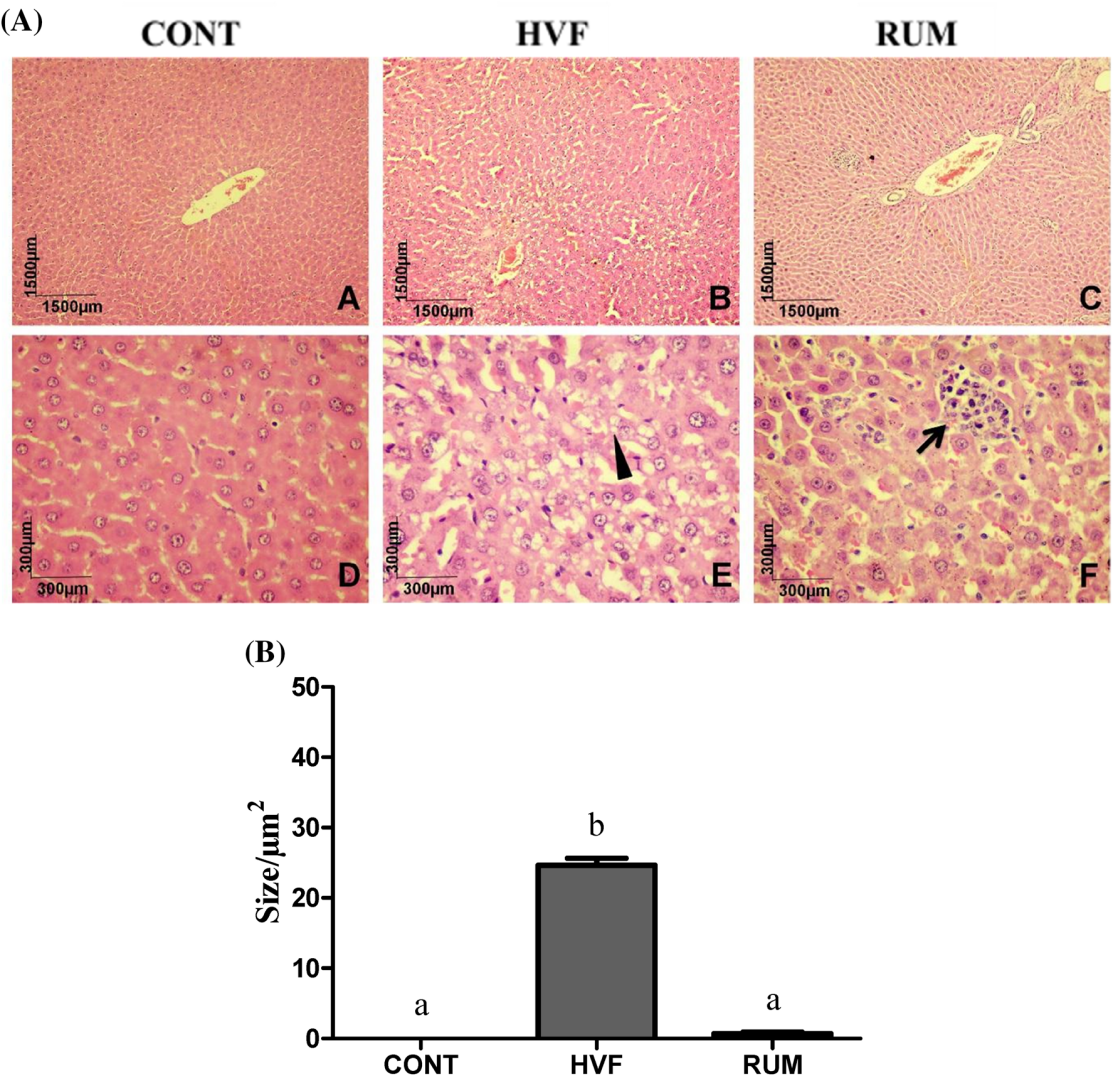
Liver histology (**A**) and histological morphometry of hepatic steatosis (**B**) of rat livers that were exposed to control diet (CONT) or diets with hydrogenated vegetable fat (HVF) or ruminant fat (RUM). Liver for rats fed CONT did not presented any steatosis lesion area (mean = 0; variance = 0) and thus were excluded from the ANOVA, that were used to compared only HVF and RUM treatments. Bars present the means and standard errors that differ significantly (*P* = 0.0001, F_(1,4)_). → Steatosis; ▲ Inflammation.

The rats fed HVF diet had livers with macroscopic lesions compatible with fatty liver syndrome (Fig. 2A), which was confirmed by the higher histological morphometry of the hepatic steatosis compared to RUM (Fig. 2B) (*P* < 0.001) and the absence of steatosis lesions in CONT. The livers of rats fed RUM diet, despite not having a fatty liver, showed milder inflammation in the histological evaluation.

### Gut microbiota composition

**Figure 3:**
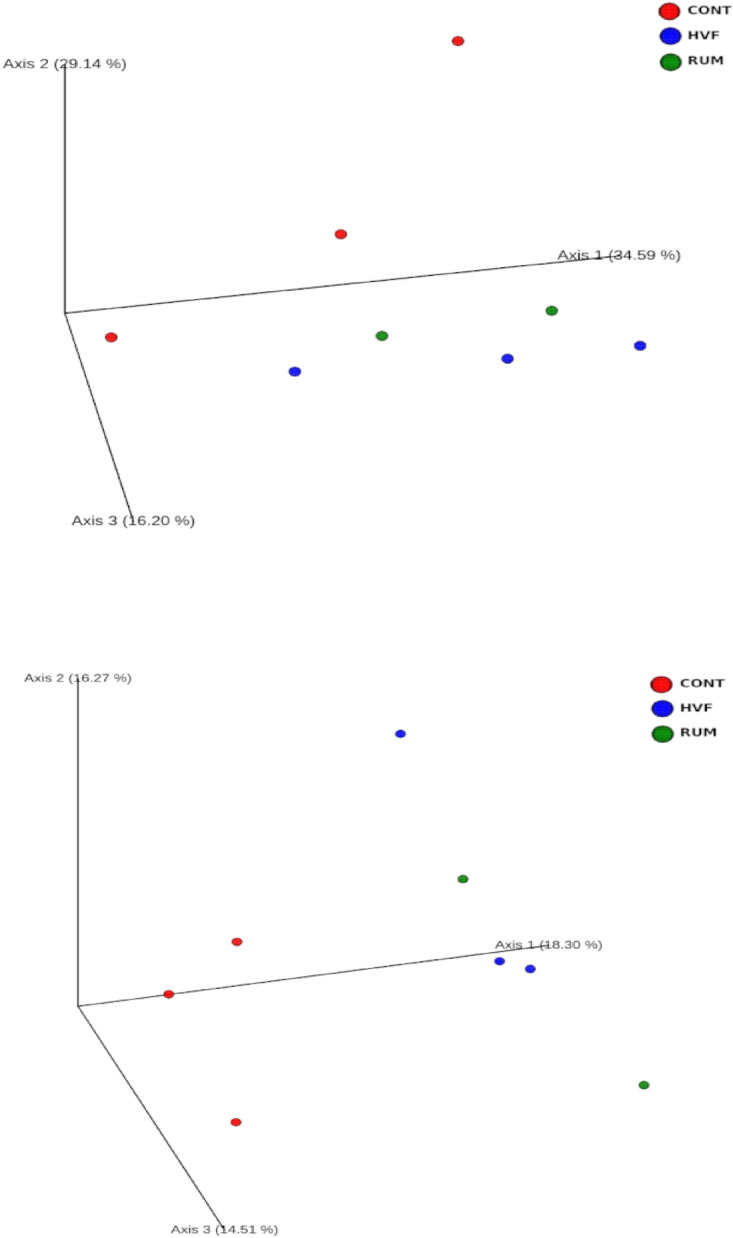
Principal coordinates analysis (PCoA) based on weighted (**A**) and unweighted-Unifrac (**B**) distance matrixes of gut microbiota composition of rats exposed to control diet (CONT) or diets with hydrogenated vegetable fat (HVF) or ruminant fat (RUM).

Rarefaction curves of observed OTUs in composed samples of the treatment groups CONT, HVF and RUM are shown in Supplementary Fig. S1. No significant differences were observed between treatments regarding richness and evenness of the gut microbial communities as evidenced by the alpha diversity indices Chao1, Shannon and Simpson (Supplementary Fig. S2). Nevertheless, the microbial gut communities differed (*P* < 0.048) in terms of beta diversity, particularly for RUM treatment (Fig. 3). Relative abundances of the most abundant phyla (Firmicutes, Bacteroidetes and Proteobacteria) and genera (*Anaerorhabdus, Clostridium, Eubacterium, Helicobacter, Prevotella, Ruminococcus, Selenomonas*) across the treatments are shown in the figures in the Supplementary material (Supplementary Fig. S3).

**Figure 4:**
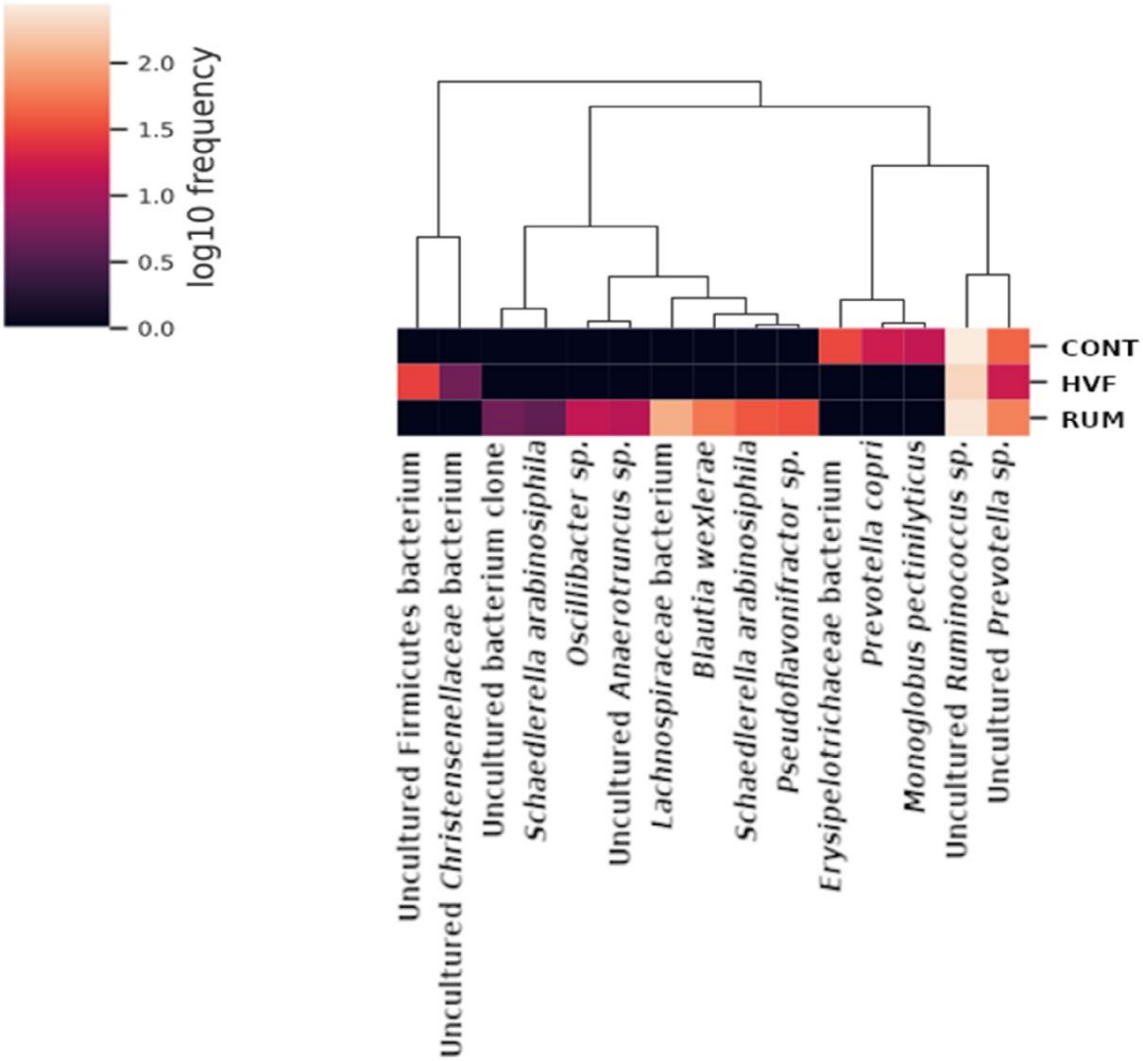
Hierarchical clustering of the machine learning classification of Amplicon Sequence Variants (ASVs) in the gut of rats exposed to control diet (CONT) or diets with hydrogenated vegetable fat (HVF) or ruminant fat (RUM). Taxonomic classification was carried out using BLASTn. The heatmap indicates abundance variations from 0 to twofold.

The differential abundance analysis revealed increased abundance of *Lachnispiraceae, Pseudoflavonifractor* and *Blautia* in the gut of rats receiving RUM diet. Ruminoccocus abundance seemed not to be affected by diets. *Prevotella* and *Monoglobus* populations were reduced in the gut of animals receiving both HVF and RUM (Fig. 4).

## Discussion

We demonstrated for the first time that the consumption of a HFD rich in iTFA or rTFA did not modify serum biochemical parameters such as blood glucose, lipoproteins, transaminases or hepatic oxidative parameters such as MDA and CAT, compared to the control group. However, iTFA reduced triglyceride and VLDL levels. In turn, rTFA intake reduced the levels of the anti-inflammatory cytokine IL-10 in blood serum, as well as hepatic steatosis, without, however, impacting greater body weight gain in rats.

These results seem to be related to the increase in the relative abundance of gut bacteria in RUM, which are inversely related to inflammation, as well as to the lower deposits of trans-fatty acids in the heart, liver and adipose tissue. Although it is well established that HFD intake causes dysbiosis and changes in metabolic parameters in rodents^10,37^, our findings suggest that not only the amount of fat in diet^10^, but also the type of fatty acids ingested and diet length will impact gut microbiota composition and some metabolic parameters^10,38^.

An important aspect to be considered in studies involving HFD is energy and lipid intake. Although the RUM and HFV groups consumed a smaller amount of feed, these groups consumed three times more lipids compared to CONT. These results have also been observed in previous studies with rats fed with HFD in the short-term^29,39^ and it may be associated with the expression of intestinal satiety-inducing peptides, such as cholecystokinin, glucagon-like peptide-1, pancreatic polypeptide, peptide YY and oxymomodulin^29,40^. However, C57/BL6 mice consumed excessively HFD over 9 weeks^41^ which may be associated with multiple feedback mechanisms, including homeostatic, hedonic and cognitive feedback in the medium and long term^41,42^.

The RUM and HVF groups had lower energy intake, although they maintained body weight gain similar to the CONT group. Together, these results indicate that the lower energy intake observed in RUM and HVF reflected greater efficiency in the conversion into weight gain, possibly due to the different amounts of carbohydrates, proteins and lipids consumed by the groups. Similar results have already been observed in rats fed HFD for five weeks^29,39,40^.

Our results showed that rats fed HVF presented lower accumulation of abdominal and epididymal adipose tissue than the CONT. This effect may be explained by the evidence, still under analysis, of the isolated effect of TFA on adipose tissue lipolysis enzymes^43^. A full understanding of the mechanisms behind this effect would require measurement of enzyme activity, protein expression as related to fatty acid mobilization, and transcription factor activity^43,44^.

Regarding serum biochemical parameters, rats fed with HVF diet presented lower serum triglycerides and VLDL than CONT and RUM. However, the glucose, total cholesterol, HDL, LDL, ALT and AST serum parameters were not modified among the dietary treatments. This favourable effect of HVF on these blood lipoproteins is surprising as the HVF diet is rich in iTFAs, as they are known for their negative effects on serum lipoprotein profile, not only in randomized, placebo-controlled clinical trials^45,46^, but also in experimental research with animals^47,48^.

On the other hand, the specific effects of *trans*-fatty acids are often difficult to discern because of other fatty acids that are present in varying amounts in both *trans*-fat and natural fat diets^47^. A previous study with Sprague–Dawley rats has already demonstrated that the metabolic effects of elaidic acid, the major isomer generated during industrial hydrogenation of oils, were modified by the types and proportions of fatty acids in the diet, mainly unsaturated fatty acids^49^. This suggests that the effects of the HVF diet found on the serum levels of triglycerides and VLDL in the present study may be associated with the source of unsaturated fatty acids rather than the presence of TFA.

A previous study with mice that consumed diets in different fatty acid compositions added with partially hydrogenated vegetable oil, evaluated the removal capability of triglycerides-rich lipoproteins by measuring lipoprotein lipase activity^50^. These researchers demonstrated that serum triglyceride levels are related to the balance between hepatic secretion and peripheral tissue clearance through lipoprotein lipase activity^50^. Therefore, the lower serum levels of triglycerides of HVF group in the present study may be related to a differential removal of triglycerides by the lipoprotein lipase enzyme in extrahepatic tissues.

Although there were no differences between groups regarding MDA content and antioxidant capacity in liver and IL-1β and TNF-α serum cytokines among the dietary treatments, the rats fed RUM diet had higher anti-inflammatory cytokine IL-10 compared to CONT, but not to HVF. In vitro studies have demonstrated an anti-inflammatory effect of ruminant fat that may somehow contribute to explaining our results. Some rTFA may decrease the production of inflammatory prostaglandins and down-regulate the TNF gene expression in endothelial cells^51^ and CLA by an increased adiponectin secretion anti-inflammatory marker^52^. Our results corroborate those observed by Bortolin et al.^53^, where serum IL-10 and liver TG levels were not correlated with adiposity index.

Dietary intake of TFA was translated directly into an increased TFA concentration in the liver, heart and adipose tissue, as also reported by Dhibi et al.^54^. The lower accumulation of adipose tissue in the HVF group suggests that our research procedures may have predisposed the rats to the onset of non-alcoholic fatty liver disease (NAFLD), as confirmed by the histopathologic analysis of hepatic tissue. The evidence from some studies suggests that TFA causes preferential fat accumulation in the liver to the detriment of adipose tissue^43,55^, which appears to compensate by stimulating de novo lipogenesis. However, the molecular mechanisms underlying this appear to be unknown^44^.

Indeed, the direct disturbance effect of TFA on liver function is so that high-TFA diets have been used to induce NAFLD experimentally in some animal models^56–58^. Moreover, high-TFA diets might induce more severe liver steatosis than diets rich in other types of fatty acids, possibly through suppressing the enzyme adipose triglyceride lipase and subsequently promoting lipid accumulation in the liver^59^. Obara et al.^60^ have further demonstrated that excessive TFA consumption induces hepatic lipogenic gene expression and non-esterified FA influx into the liver, with the hepatic accumulation of lipid peroxide and local cytokines by Kupffer cells.

On the other hand, although RUM-fed rats showed mild inflammation in the histopathological evaluation of the liver, RUM treatment did not trigger negative metabolic changes, as discussed above. In fact, the RUM treated animals did not present as many fatty liver lesions as those from those fed HVF. Some caution, however, is needed in the interpretation of the HVF effects on fatty liver lesions, as the FA accumulation in the liver was not detected. In fact, the liver FA concentration remained normal and similar among the three treatments.

The much lower incorporation of TFA in the liver observed with RUM than with HVF could explain why the rats fed RUM did not develop the HFD-induced NAFLD. Vaccenic acid (18:1*t*11) was the overwhelming *trans*-octadecenoate present in the lamb fat used in the RUM diet. Vaccenic acid is usually the major TFA of edible ruminant fat, although, in ruminants kept under intensive feeding conditions, the 18:1*t*10 often replaces the 18:1*t*11 as the predominant TFA^3^. The 18:1*t*11 is also actively desaturated in mammal tissues by stearoyl-CoA desaturase (SCD) into 18:2*c*9,*t*11, the major CLA isomer present in ruminant fat^3^. Both 18:1*t*11 and 18:2*c*9,*t*11 have been reported to reduce hepatic lipogenesis^61^. In fact, despite being a TFA, the 18:1*t*11 seems very effective in attenuating complications observed in the metabolic syndrome in rats^62^, increasing the levels of IL-10 in adipose tissue^63^ and preventing hepatic lipid accumulation^64^.

Liver and the other tissues of the rats fed RUM also had consistently more iso- and anteiso-BCFA, and 18:0 than HVF and CONT. Thus, we can hypothesise that some of these FA might have protective effects on the liver. Indeed, one study showed that a stearic acid-rich diet combined with cell therapy accelerated the recovery of hepatic dysfunction in a rat model of liver injury^65^. However, further experimental studies are needed to assess the individual metabolic effects of BCFA and 18:0.

Evidence has been put forward to suggest that the gut microbiota is associated with liver abnormalities^66^, via the gut-liver axis. This has been confirmed under our experimental conditions: i.e., diets with 23% of fat fed over 53 days with the RUM treatment seemed to be more effective in reshaping the gut microbiota than HVF, as suggested by the significant group dissimilarities. It is well established that the HFD intake, especially for long periods, can induce gut dysbiosis in rodents^37^.

A meta-analysis demonstrated that the HFD intake in the range of 27.1 to 65% fat might induce reproducible shifts in the rat gut microbiota, such as changes in the Firmicutes/Bacteroidetes ratio^67^. Depletion of *Blautia* and *Lachnospiraceae* has been frequently reported in rats under systemic inflammation conditions^68,69^. Therefore, the increased abundances of these bacteria observed in the gut of rats receiving RUM could be associated with the highest levels of IL-10 which is an anti-inflammatory cytokine. These results warrant further investigations on functional approaches addressing the effects of *Blautia* and *Lachnospiraceae* organisms on the systemic metabolism. The reduction of fibrolytic bacteria such as *Monoglobus* and *Prevotella* caused by the HFD intake corroborate previous findings^70,71^ and suggest that long-term use of HFD may pose undesirable changes in the gut microbiota composition of rats.

Although our study revealed that naturally and industrially produced TFAs might exert distinct effects on the gut microbiome, the microbiota composition analysis was performed using a limited number of samples. We were, despite this, able to obtain a very satisfactory number of reads per sample, as observed in the rarefaction curves, providing sufficient depth to support the detection of a putative dissimilarity between treatments by the a permutational multivariate analysis of variance (PERMANOVA). In this sense, other approaches, such as shot-gun metagenomics and metabolomics, could also be used to generate comprehensive functional information. This was not possible with the amplicon sequencing approach (metagenomic ID) used in the present study.

Another limitation of our study is that we did not assess the body composition of rats, and it is not possible to know which body component was altered to compensate for the decrease in adipose tissue in the HFV and still maintain the same body weight in all groups. However, considering that cardiovascular disease (CVD) is the leading global cause of death in Western countries, and its development is associated with unhealthy dietary patterns^72^, evaluation of the influence of different types of HFD on metabolic aspects related to cardiovascular health is still crucial^73^.

An exact comparison of our results with the outcomes of HFD intake reported in previous studies may not be reliable, considering the quantity and diversity of ingredients and, consequently, the dietary fatty acid profile and administration time. Previous studies have shown that consumption of HFD (31% kcal from lipids) containing soy oil, lard, hydrogenated vegetable fat and synthetic cholesterol as fat sources for 5 weeks was able to induce dyslipidaemia with alteration of all lipoproteins and increase in triglycerides and reduction in *Lactobacillus* and *Bifidobacterium* counts, recognized as beneficial bacteria in the gut microbiota^29,39^. On the other hand, long-term consumption of a HFD containing lard and soybean oil and 60% kcal from lipids for 18 weeks did not strongly affect serum lipid profile or levels of alanine aminotransferase and aspartate aminotransferase nor the gut microbiota of Wistar rats^53^. This might have been a compensatory response caused by the chronic HFD intake, not yet elucidated^74^.

In humans the sustained adherence to a HFD appears to be difficult in the long-term. There is a lack of data to support efficacy, safety, and health benefits of this diet’s intake^75^. It is the position of the Academy of Nutrition and Dietetics that dietary fat for the healthy adult population should provide 20% to 35% of energy, with a limitation on *trans* fats from industrial sources–iTFA^76^. Even in high-fat diets, the amount of natural trans fats consumed in the human diet is small.

Future translational research on the consumption of natural ruminant fat evaluating the effects of rTFA in the population can be a great challenge, considering that in the human diet fatty acids are consumed as a part of foods that contain other nutrients and dietary compounds; these can have additive and synergistic effects in health and interact in complex ways that would be difficult to delineate^77^. Our study design demonstrates the short-term outcomes of iTFA compared to rTFA intake and may contribute to future studies that work with food matrices elaborated with the respective types of *trans*-fatty acids that, in turn, may contribute to public health and food technology.

## Conclusion

Dietary TFA sources (industrial *vs* ruminant natural) altered some metabolic parameters, TFA deposits in tissues and gut microbiota composition in rats without, however, changing their body weight gain. The lower levels of triglycerides and VLDL and the lower adiposity presented by rats fed with iTFA did not impact the improvement of other parameters in the gut-liver axis that were evaluated. However, it is remarkable that ruminant fat reversed the hepatic steatosis normally caused by high fat diets, which may be related to the remodelling of the gut microbiota and its potential anti-inflammatory activity.

## Supplementary Information

Supplementary Information.

## Data Availability

The datasets generated during and/or analyzed during the current study are available from the corresponding author upon reasonable request.

## References

[1] Cascio G, Schiera G, Di Liegro I (2011). Dietary fatty acids in metabolic syndrome, diabetes and cardiovascular diseases. Curr. Diabetes Rev..

[2] Takeuchi H, Sugano M (2017). Industrial Trans fatty acid and serum cholesterol: the allowable dietary level. J. Lipids.

[3] Bessa RJB, Alves SP, Santos-Silva J (2015). Constraints and potentials for the nutritional modulation of the fatty acid composition of ruminant meat. Eur. J. Lipid Sci. Technol..

[4] Mapiye C (2015). The trans-octadecenoic fatty acid profile of beef: implications for global food and nutrition security. Food Res. Int..

[5] Aldai N, de Renobales M, Barron LJR, Kramer JKG (2013). What are the *trans* fatty acids issues in foods after discontinuation of industrially produced *trans* fats? Ruminant products, vegetable oils, and synthetic supplements. Eur. J. Lipid Sci. Technol..

[6] Dugan MER, Aldai N, Aalhus JL, Rolland DC, Kramer JKG (2011). Review: trans-forming beef to provide healthier fatty acid profiles. Sci. Rep..

[7] Wang DD, Hu FB (2017). Dietary fat and risk of cardiovascular disease: recent controversies and advances. Annu. Rev. Nutr..

[8] Hammad S, Pu S, Jones PJ (2016). Current evidence supporting the link between dietary fatty as and cardiovascular disease. Lipids.

[9] Kuhnt K, Degen C, Jahreis G (2016). Evaluation of the impact of ruminant *trans* fatty acids on human hcidealth: important aspects to consider. Crit. Rev. Food Sci. Nutr..

[10] Ge Y (2019). Effect of industrial trans-fatty acids-enriched diet on gut microbiota of C57BL/6 mice. Eur. J. Nutr..

[11] Hua Y (2020). Trans-fatty acids alter the gut microbiota in high-fat-diet-induced obese rats. Br. J. Nutr..

[12] Roy A (2007). Butters rich either in trans-10-C18:1 or in trans-11-C18:1 plus cis-9, trans-11 CLA differentially affect plasma lipids and aortic fatty streak in experimental atherosclerosis in rabbits. Animal.

[13] de Almeida MM (2015). Cis-9, trans-11 and trans-10, cis-12 CLA Mixture does not change body composition, induces insulin resistance and increases serum hdl cholesterol level in rats. J. Oleo Sci..

[14] Brouwer IA, Wanders AJ, Katan MB (2013). Trans fatty acids and cardiovascular health: research completed?. Eur. J. Clin. Nutr..

[15] Décarie-Spain L (2018). Nucleus accumbens inflammation mediates anxiodepressive behavior and compulsive sucrose seeking elicited by saturated dietary fat. Mol. Metab..

[16] Albillos A, Gottardi A, Rescigno M (2020). The gut-liver axis in liver disease: Pathophysiological basis for therapy. J Hepatol..

[17] WHO | World Health Organization. Diet, nutrition and the prevention of chronic diseases. *WHO* (2014).

[18] WHO | World Health Organization. A comprehensive global monitoring framework including indicators and a set of voluntary global targets for the prevention and control of non communicabale diseases, *WHO* (2012).

[19] Ministério da Saúde. http://bvsms.saude.gov.br/bvs/saudelegis/anvisa/2003/rdc0360_23_12_2003.html.

[20] Nagy, K. & Tiuca, I.-D. Importance of fatty acids in physiopathology of human body. *Fatty Acids* (InTech, 2017). 10.5772/67407.

[21] Müller CP (2015). Brain membrane lipids in major depression and anxiety disorders. Biochim Biophys Acta..

[22] Speakman JR (2019). Use of high-fat diets to study rodent obesity as a model of human obesity. Int J Obes.

[23] Reeves PG, Nielsen FH, Fahey GC (1993). AIN-93 purified diets for laboratory rodents: final report of the American Institute of Nutrition ad hoc writing committee on the reformulation of the AIN-76A rodent diet. J. Nutr..

[24] du Sert, N. P. *et al*. The ARRIVE guidelines 2.0: updated guidelines for reporting animal research. *Plos Biol*, **18** e3000410 (2020). 10.1371/journal.pbio.3000410 PMC7360023 32663219

[25] Tuck MK (2009). Standard operating procedures for serum and plasma collection: early detection research network consensus statement standard operating procedure integration working group. J. Proteome Res..

[26] Alves, S. P., Raundrup, K., Cabo, Â., Bessa, R. J. B. & Almeida, A. M. Fatty acid composition of muscle, adipose tissue and liver from muskoxen (Ovibos moschatus) living in West Greenland. *PLoS One* **10**, e0145241 (2015). 10.1371/journal.pone.0145241 PMC4683068 26678792

[27] Winterbourn CC, Gutteridge JMC, Halliwell B (1985). Doxorubicin-dependent lipid peroxidation at low partial pressures of O2. J. Free Radicals Biol. Med..

[28] Brand-Williams, W., Cuvelier, M. E. & Berset, C. Use of a free radical method to evaluate antioxidant activity. **28** (1995).

[29] Batista KS (2018). Beneficial effects of consumption of acerola, cashew or guava processing by-products on intestinal health and lipid metabolism in dyslipidaemic female Wistar rats. Br. J. Nutr..

[30] Bolyen E (2019). Reproducible, interactive, scalable and extensible microbiome data science using QIIME 2. Nat. Biotechnol..

[31] Callahan BJ (2016). DADA2: high-resolution sample inference from Illumina amplicon data. Nat. Methods.

[32] Janssen, S. *et al.* Phylogenetic placement of exact amplicon sequences improves associations with clinical information. *mSystems* **3**, (2018). 10.1128/mSystems.00021-18 PMC5904434 29719869

[33] McMurdie, P. J. & Holmes, S. phyloseq: An R Package for reproducible interactive analysis and graphics of microbiome census data. *PLoS One* **8**, e61217 (2013). 10.1371/journal.pone.0061217 PMC3632530 23630581

[34] McDonald D (2012). An improved Greengenes taxonomy with explicit ranks for ecological and evolutionary analyses of bacteria and archaea. ISME J..

[35] Milliken, G. A. & Johnson, D. E. Analysis of messy data, Volume I: *Analysis of designed experiments.* 2nd ed. (Chapman and Hall, 2009).

[36] Gish W, Altschul SF, Lipman DJ, Miller W, Myers EW (1990). Basic local alignment search tool. J. Mol. Biol..

[37] He, C. *et al.* High-fat diet induces dysbiosis of gastric microbiota prior to Gut microbiota in association with metabolic disorders in mice. *Front. Microbiol.* **9** (2018). 10.3389/fmicb.2018.00639 PMC5900050 29686654

[38] Buettner R, Schölmerich J, Bollheimer LC (2007). High-fat diets: modeling the metabolic disorders of human obesity in rodents. Obesity.

[39] Bezerra, M. L. R. *et al*. Effects of honey from Mimosa quadrivalvis L. (malicia) produced by the Melipona subnitida D. (jandaira) stingless bee on dyslipidaemic rats. *Food Funct.* **9**, 4480–4492 (2018). 10.1039/c8fo01044g 30080211

[40] Shirazi., R. *et al.* Glucagon-like peptide 1 receptor induced suppression of food intake, and body weight is mediated by central IL-1 and IL-6. *Proc. Natl. Acad. Sci. U. S. A*. **110** 16199–16204 (2013). 10.1073/pnas.1306799110 PMC3791711 24048027

[41] Licholai JA (2018). Why do mice over-eat high fat diets? How high fat diet alters the regulation of daily caloric intake in mice. Obesity.

[42] Hall KD, Hammond RA, Rahmandad H (2014). Dynamic interplay among homeostatic, hedonic, and cognitive feedback circuits regulating body weight. Am. J. Public Health..

[43] Machado RM (2010). Intake of trans fatty acids causes nonalcoholic steatohepatitis and reduces adipose tissue fat content. J. Nutr..

[44] Oteng, A-B. & Kersten, S. Mechanisms of action of trans fatty acids. *Adv. Nutr*. **11** 697–708 (2020). 10.1093/advances/nmz125 PMC7231579 31782488

[45] Aronis KN, Khan SM, Mantzoros CS (2012). Effects of trans fatty acids on glucose homeostasis: a meta-analysis of randomized, placebo-controlled clinical trials. Am. J. Clin. Nutr..

[46] Mozaffarian D, Clarke R (2009). Quantitative effects on cardiovascular risk factors and coronary heart disease risk of replacing partially hydrogenated vegetable oils with other fats and oils. Eur. J. Clin. Nutr..

[47] Wijendran V, Pronczuka A, Bertolib C, Hayes KC (2003). Dietary trans-18:1 raises plasma triglycerides and VLDL cholesterol when replacing either 16:0 or 18:0 in gerbils. J. Nutr. Biochem..

[48] Longhi R (2017). Effect of a trans fatty acid-enriched diet on biochemical and inflammatory parameters in Wistar rats. Eur. J. Nutr..

[49] Koga T, Nonaka M, Gu JY, Sugano M (1997). Linoleic and alpha-linolenic acids differently modify the effects of elaidic acid on polyunsaturated fatty acid metabolism and some immune indices in rats. Br. J. Nutr..

[50] Saín J, González MA, Lavandera JV, Scalerandi MV, Bernal CA (2016). The effects of trans-fatty acids on TAG regulation in mice depend on dietary unsaturated fatty acids. Br. J. Nutr..

[51] Da Silva MS (2017). Modulation of the biomarkers of inflammation and oxidative stress by ruminant trans fatty acids and dairy proteins in vascular endothelial cells (HUVEC). Prostaglandins Leukot. Essent. Fat. Acids.

[52] Ahn IS (2006). Isomer-specific effect of conjugated linoleic acid on inflammatory adipokines associated with fat accumulation in 3T3-L1 adipocytes. J. Med. Food.

[53] Bortolin RC (2018). A new animal diet based on human Western diet is a robust diet-induced obesity model: comparison to high-fat and cafeteria diets in term of metabolic and gut microbiota disruption. Int. J. Obes..

[54] Dhibi M (2016). Consumption of oxidized and partially hydrogenated oils differentially induces trans-fatty acids incorporation in Rats’ heart and dyslipidemia. J. Am. Coll. Nutr..

[55] Oteng, A-B., Loregger, A., van Weeghel, M., Zelcer, N. & Kersten S. Industrial trans fatty acids stimulate SREBP2-mediated cholesterogenesis and promote non-alcoholic fatty liver disease. *Mol. Nutr. Food Res.* **63**, e1900385 (2019). 10.1002/mnfr.201900385 PMC6790681 31327168

[56] Liu WH, Lin CC, Wang ZH, Mong MC, Yin MC (2010). Effects of protocatechuic acid on trans fat induced hepatic steatosis in mice. J. Agric. Food Chem..

[57] Santos JDB (2018). Food-drug interaction: anabolic steroids aggravate hepatic lipotoxicity and non-alcoholic fatty liver disease induced by trans fatty acids. Food Chem. Toxicol..

[58] Komatsu G (2019). AIM-deficient mouse fed a high-trans fat, high-cholesterol diet: a new animal model for non-alcoholic fatty liver disease. Exp. Anim..

[59] Zhao, X. *et al.* Trans-fatty acids aggravate obesity, insulin resistance and hepatic steatosis in C57BL/6 mice, possibly by suppressing the IRS1 dependent pathway. *Molecules* **21** (2016). 10.3390/molecules21060705 PMC6273562 27248994

[60] Obara N (2010). Possible involvement and the mechanisms of excess trans-fatty acid consumption in severe NAFLD in mice. J. Hepatol..

[61] Jacome-Sosa MM (2010). Increased hypolipidemic benefits of cis-9, trans-11 conjugated linoleic acid in combination with trans-11 vaccenic acid in a rodent model of the metabolic syndrome, the JCR:LA-cp rat. Nutr. Metab..

[62] Wang Y (2009). Trans-11 vaccenic acid reduces hepatic lipogenesis and chylomicron secretion in JCR:LA-cp rats. J. Nutr..

[63] Mohankumar SK (2013). Dietary supplementation of trans-11-vaccenic acid reduces adipocyte size but neither aggravates nor attenuates obesity-mediated metabolic abnormalities in fa/fa Zucker rats. Br. J. Nutr..

[64] Jacome-Sosa MM (2014). Diets enriched in trans-11 vaccenic acid alleviate ectopic lipid accumulation in a rat model of NAFLD and metabolic syndrome. J. Nutr. Biochem..

[65] Goradel NH (2016). Improvement of liver cell therapy in rats by dietary stearic acid. Iran. Biomed. J..

[66] Fan SX (2021). Mechanism of gut-microbiota-liver axis in the pathogenesis of intestinal failure-associated liver disease. Zhonghua Wei Chang Wai Ke Za Zhi.

[67] Bisanz JE, Upadhyay V, Turnbaugh JA, Ly K, Turnbaugh PJ (2019). Meta-analysis reveals reproducible Gut microbiome alterations in response to a high-fat diet. Cell Host Microbe.

[68] Zhu Y (2019). Gut microbiota dysbiosis worsens the severity of acute pancreatitis in patients and mice. J. Gastroenterol..

[69] Zheng, J. *et al.* Improvement on metabolic syndrome in high fat diet-induced obese mice through modulation of gut microbiota by Sangguayin decoction. *J. Ethnopharmacol.* **246** (2020). 10.1016/j.jep.2019.112225 31509781

[70] Chen T (2017). Fiber-utilising capacity varies in Prevotella- versus Bacteroides-dominated gut microbiota. Sci. Rep..

[71] Kim CC (2019). Genomic insights from Monoglobus pectinilyticus: a pectin-degrading specialist bacterium in the human colon. ISME J..

[72] Carro A, Panisello JM (2019). Deciphering the Riddles in Nutrition and Cardiovascular Disease. Eur. Cardiol. Rev..

[73] Crescenzo R (2015). Fat quality influences the obesogenic effect of high fat diets. Nutrients.

[74] Ströher DJ (2020). Virgin coconut oil associated with high-fat diet induces metabolic dysfunctions, adipose inflammation, and hepatic lipid accumulation. J. Med. Food..

[75] Brouns F (2018). Overweight and diabetes prevention: is a low-carbohydrate–high-fat diet recommendable?. Eur. J. Nutr..

[76] Remig V (2010). Trans fats in America: a review of their use, consumption, health implications, and regulation. J. Am. Diet Assoc..

[77] Vannice G, Rasmussen H (2014). Position of the academy of nutrition and dietetics: dietary fatty acids for healthy adults. J. Acad. Nutr. Diet..

