# Supplementary material for: Ruminant fat intake improves gut microbiota, serum inflammatory parameter and fatty acid profile in tissues of Wistar rats
Source: Sci Rep. 2021 Sep 23;11:18963. doi: 10.1038/s41598-021-98248-6 (PMC8460723; doi:10.1038/s41598-021-98248-6)
Supplement: Supplementary file 1 — Supplementary Information. [file 41598_2021_98248_MOESM1_ESM.docx]

**Supplementary Material**


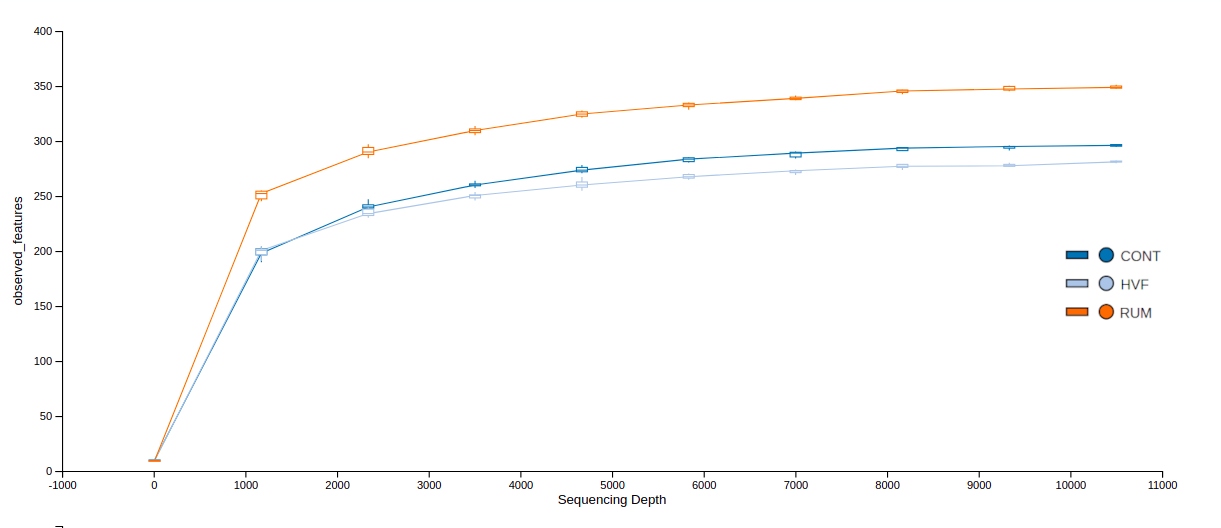


**Supplementary Figure S1-** Rarefaction curves of observed OTUs in the samples of the three treatment groups CONT, HVF and RUM.

**A)**


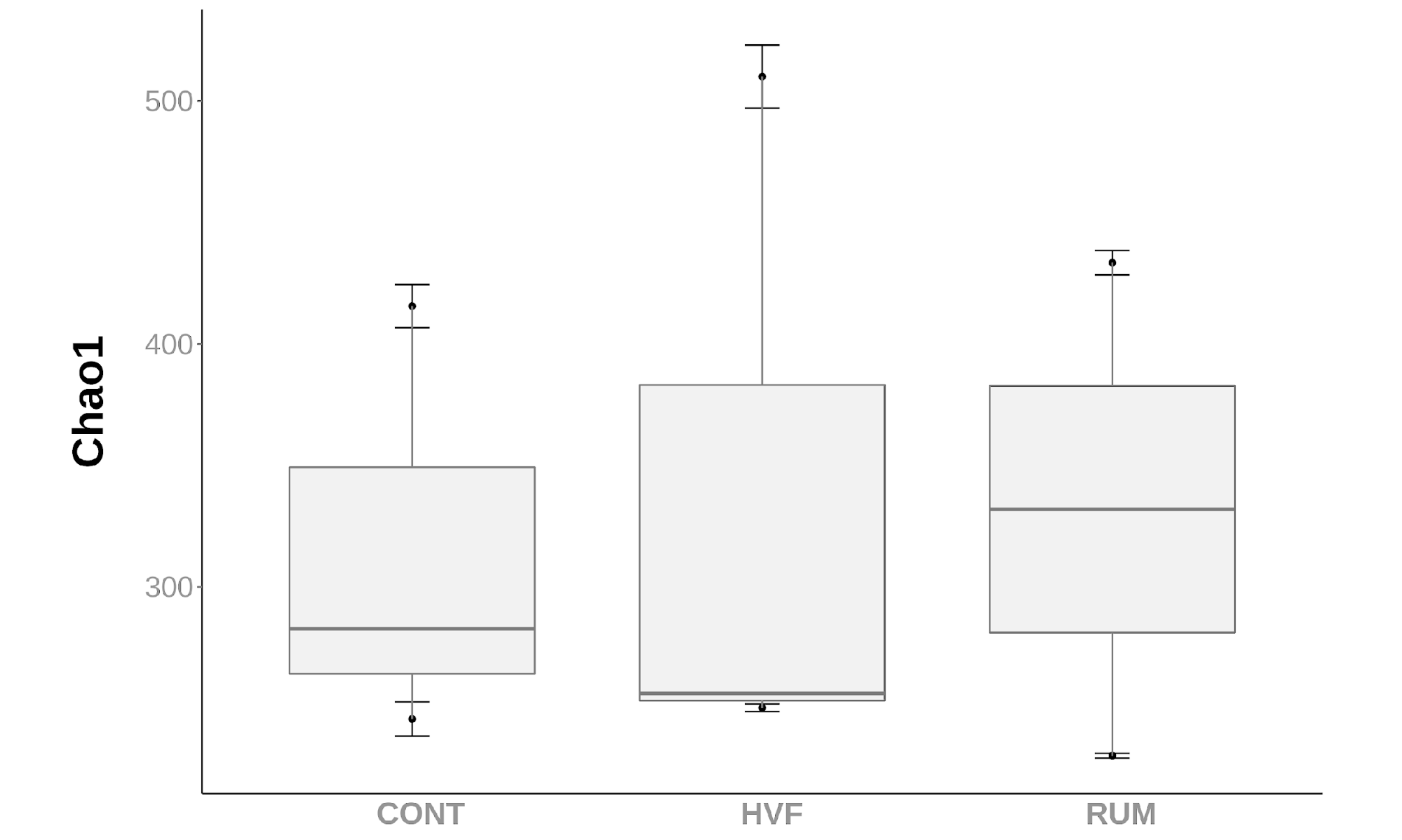


**B)**


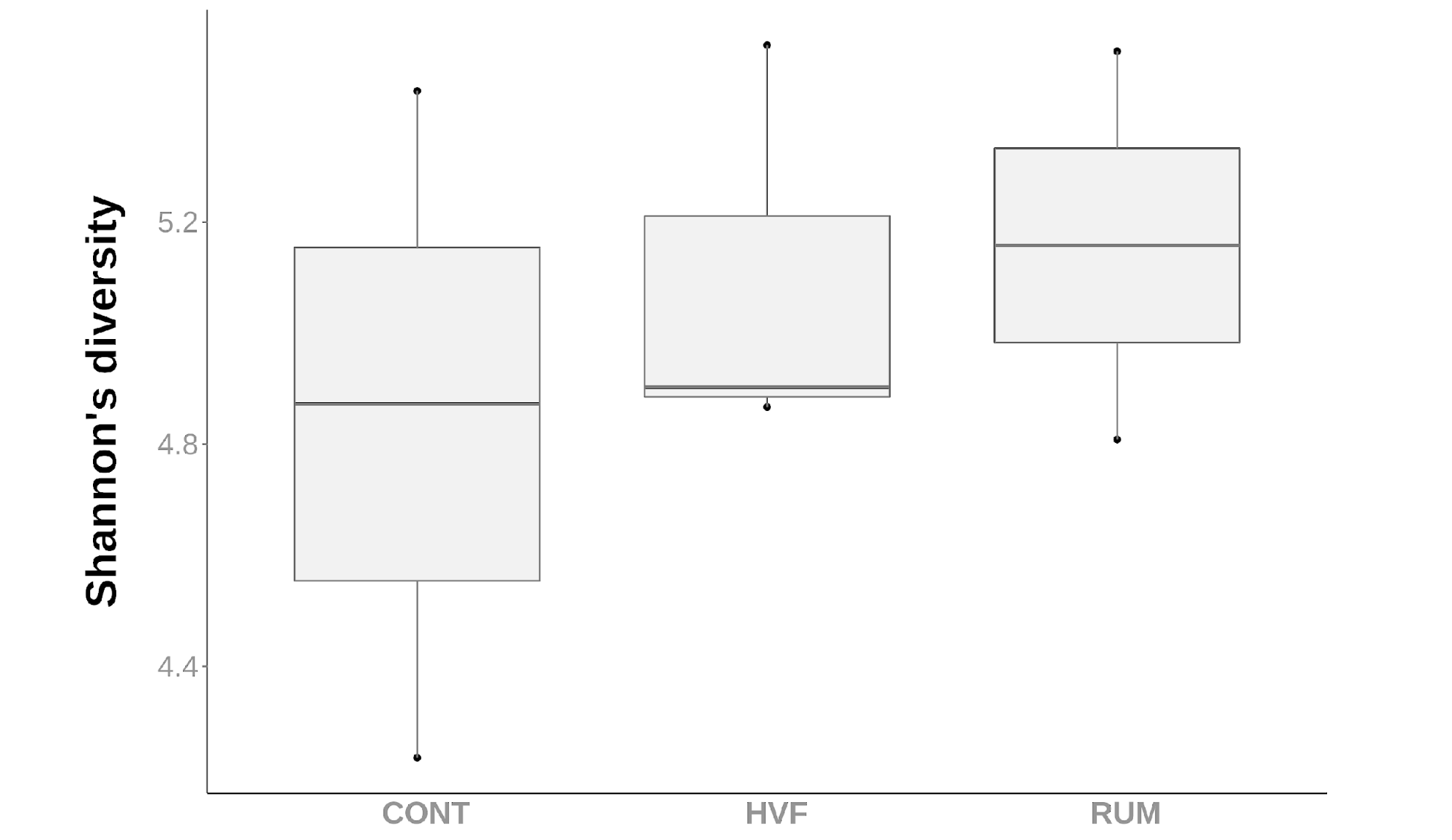


**C)**


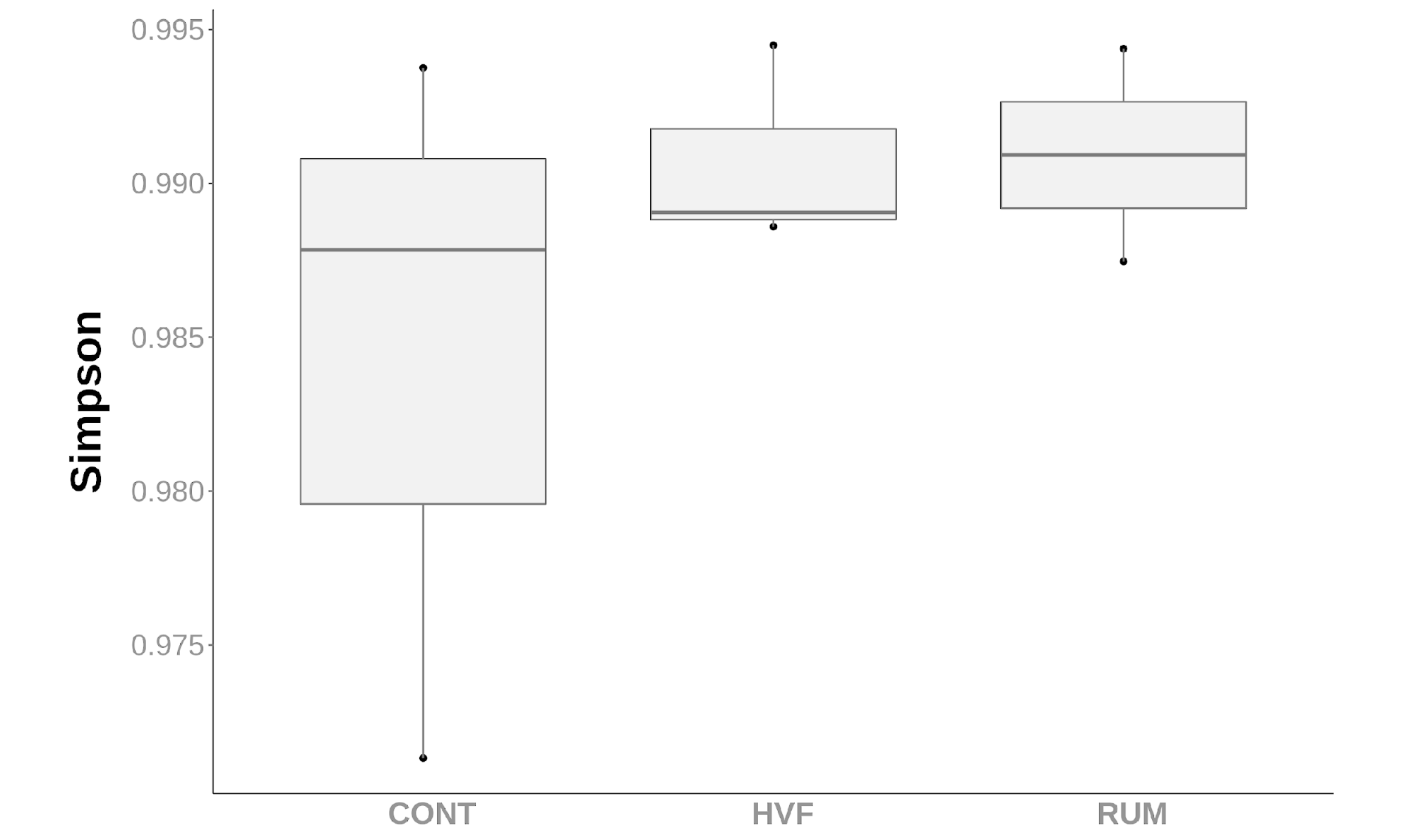


**Supplementary Figure S2** – Alpha-diversity measured by the diversity indices Chao1 (A), Simpson (B) and Shannon (C) of the gut microbiota of rats exposed to control diet (CONT) or diets supplemented with hydrogenated vegetable fat (HVF) or ruminant fat (RUM).

**A)**


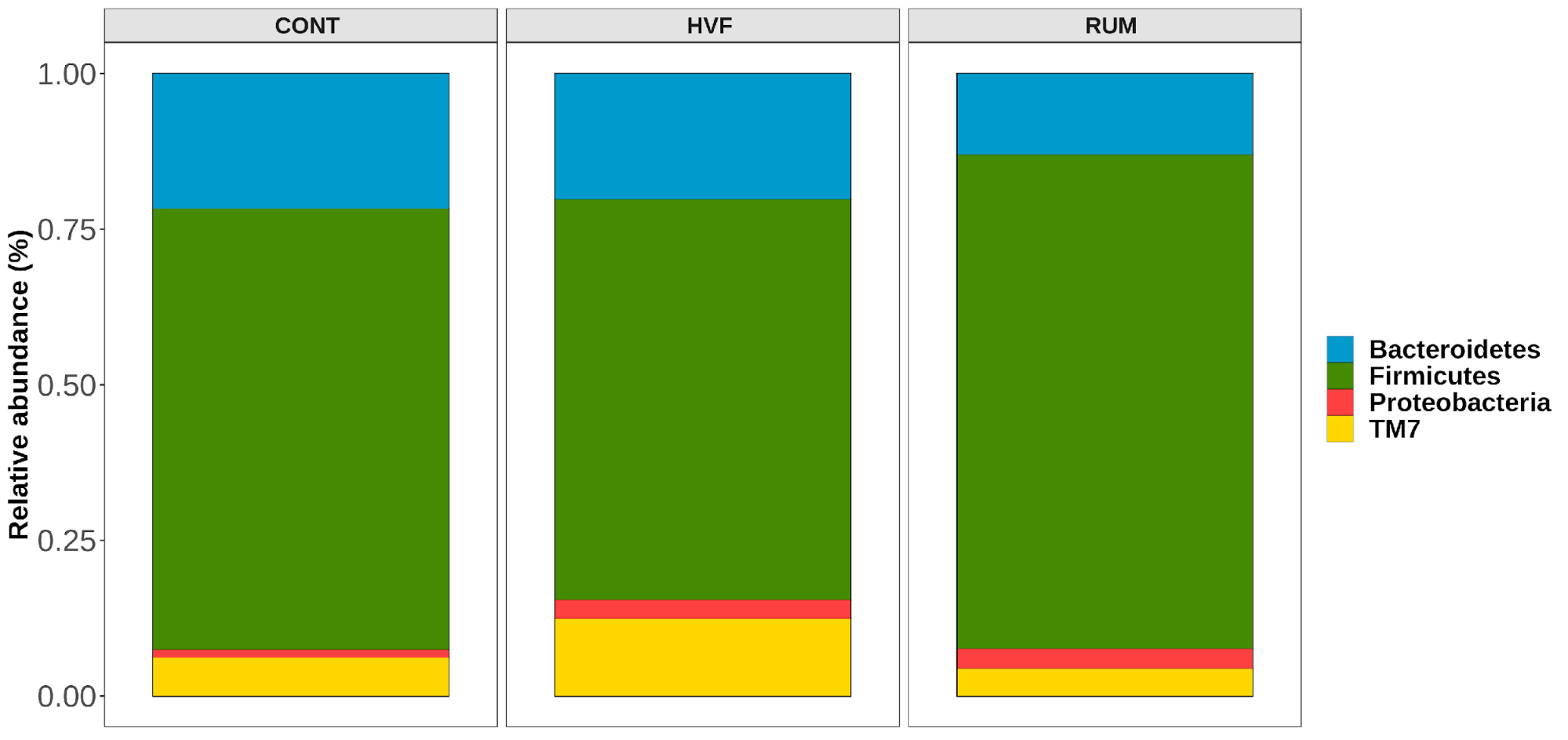


**B)**
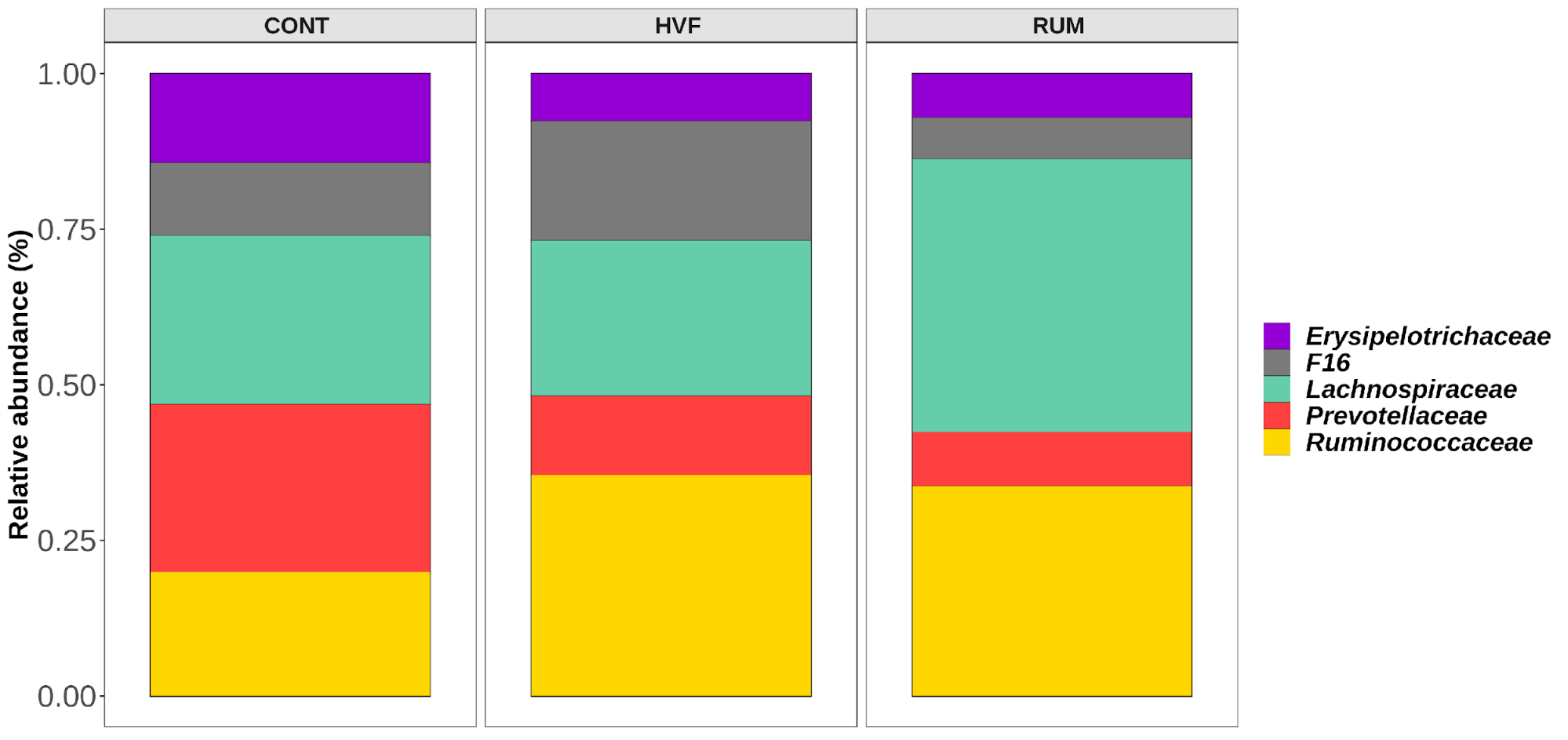


**C)**


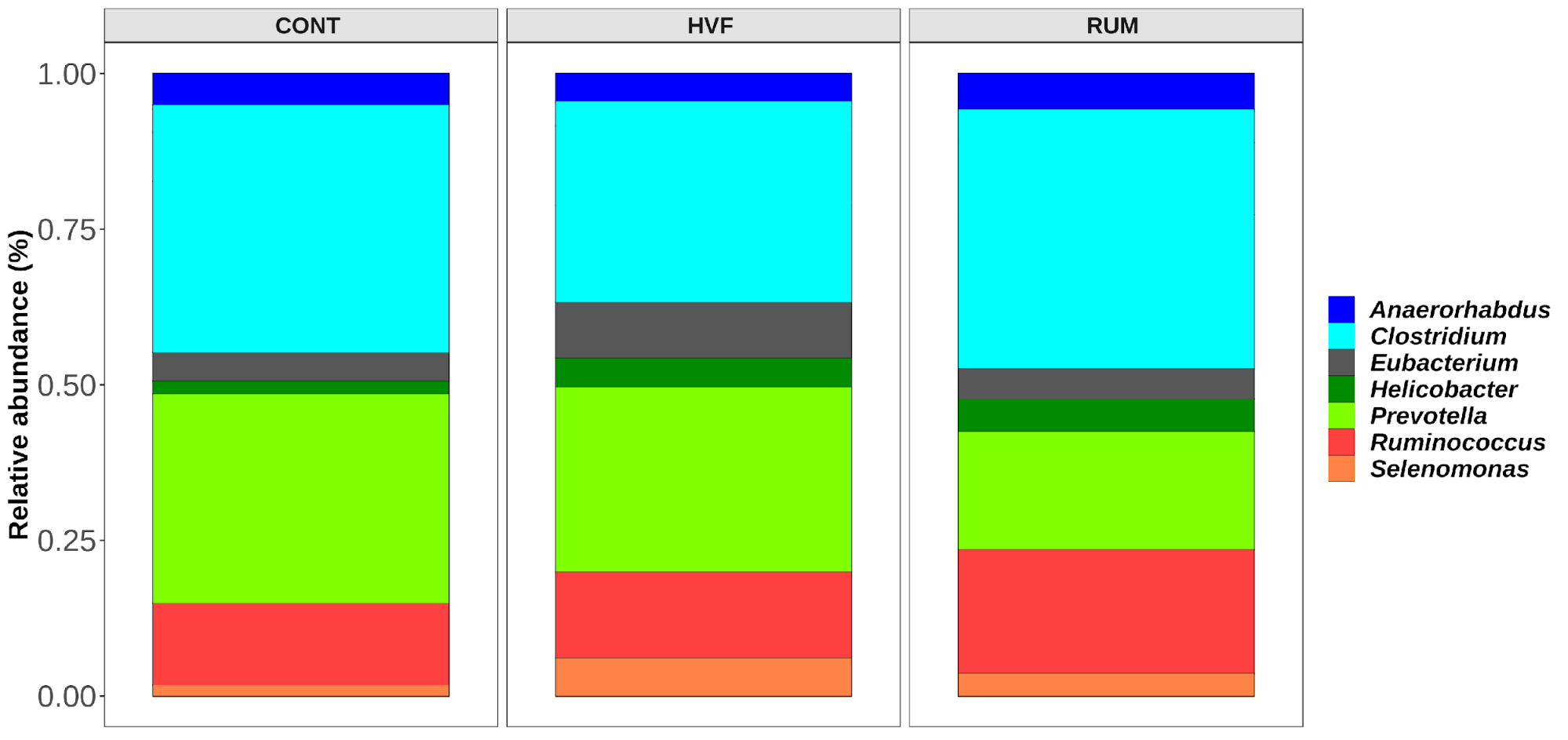


**Supplementary Figure S3** - Relative abundance of microbial taxa at phylum (A), family (B) and genus (C) levels in the gut of rats exposed to control diet (CONT) or diets supplemented with hydrogenated vegetable fat (HVF) or ruminant fat (RUM).

**Table S1** – Proximate composition and energy content of control diet (CONT) and diets with the addition of hydrogenated vegetable fat (HVF) or ruminant fat (RUM).

| Proximate composition and energy content/ 100 g of diet | Diets | | |
| --- | --- | --- | --- |
|  | **CONT** | **HVF** | **RUM** |
| Moisture (%) | 10.46 | 14.26 | 13.10 |
| Ash (%) | 5.86 | 6.03 | 5.76 |
| Carbohydrates (%) | 59.61 | 41.58 | 33.11 |
| Proteins (%) | 18.84 | 16.69 | 24.04 |
| Lipids (%) | 5.23 | 21.44 | 23.99 |
| Energy value (kcal) | 360.87 | 426.04 | 444,51 |
| Carbohydrates (kcal %) | 66.07 | 39.04 | 29.79 |
| Proteins (kcal %) | 20.88 | 15.67 | 21.63 |
| Lipids (kcal %) | 13.04 | 45.29 | 48.57 |

CONT: Control; HVF: hydrogenated vegetable fat; RUM: ruminant fat.

**Table S2 –** Short notation, systematic and common names of fatty acids listed in the tables, ordered by elution order.

| **Short notation** | **Systematic name** | **Common name** |
| --- | --- | --- |
| 14:0 | tetradecanoic acid | myristic acid |
| 15:0 | pentadecanoic acid | pentadecylic acid |
| i-16:0 | 14-methylpentadecanoic acid | isopalmitic acid |
| 16:0 | hexadecanoic acid | palmitic acid |
| 16:1-*t* | undefined *trans*-hexadecenoic acid | - |
| i-17:0 | 15-methylhexadecanoic acid | isomargaric acid |
| 16:1-*c*7 | *cis*-7-hexadecenoic acid | - |
| 16:1-*c*9 | *cis*-9-hexadecenoic acid | palmitoleic acid |
| a-17:0 | 14-methylhexadecanoic acid | anteisomargaric acid |
| 17:0 | heptadecanoic acid | margaric acid |
| i-18:0 | 16-methylheptadecanoic acid | isostearic acid |
| 17:1-*c*9 | *cis*-9-heptadecenoic acid | - |
| 18:0 | Octadecanoic acid | stearic acid |
| 18:1-*t*6/-*t*7/-*t*8 | Coeluted peak of trans-6- octadecenoic, trans-7- octadecenoic and trans-8-octadecenoic acids | - |
| 18:1-*t*9 | *trans*-9-octadecenoic acid | elaidic acid |
| 18:1-*t*10 | *trans*-10-octadecenoic acid | - |
| 18:1-*t*11 | *trans*-11-octadecenoic acid | vaccenic acid |
| 18:1-*t*12 | *trans*-12-octadecenoic acid | - |
| 18:1-*c*9 | *cis*-9-octadecenoic acid | oleic acid |
| 18:1-*t*15 | *trans*-15-octadecenoic acid | - |
| 18:1-*c*11 | *cis*-11-octadecenoic acid | asclepic acid *or cis*‐vaccenic acid |
| 18:1-*c*12 | *cis*-12-octadecenoic acid | - |
| 18:1-*c*13 | *cis*-13-octadecenoic acid | - |
| 18:1-*t*16/-*c*14 | coeluted peak of trans-16- octadecenoic acid and cis-15-octadecenoic acid | - |
| 18:1-*c*15 | *cis*-15-octadecenoic acid | - |
| 18:2oi | sum of several *trans*,*trans*-, *cis*,*trans*- or *trans*,*cis*- octadecadienoic acids | - |
| 18:2n-6 | *cis*-9,cis-12-octadecadienoic acid | linoleic acid |
| 20:0 | eicosanoic acid | arachidic acid |
| 18:3n-6 | *cis*-6, *cis*-9, *cis*-12-octadecatrienoic acid | γ-linolenic acid |
| 20:1 | Undefined eicosenoic acid isomer | - |
| 18:3n-3/20:1-*c*11 | Coeluted peak of *cis*-9, *cis*-12, *cis*-15-octadecatrienoic acid and cis-11 eicosenoic acis | α-linolenic acid/ gondoic acid |
| 18:2-*c*9*t*11 | *cis*-9,*trans*-11-octadecadienoic acid | rumenic acid |
| 20:2n-6 | *cis*-11,*cis*-14-eicosadienoic acid | dihomolinoleic acid |
| 20:3n-9 | *cis*-5, *cis*-8, *cis*-11-eicosatrienoic acid | mead acid |
| 22:0 | Docosanoic acid | behenic acid |
| 20:3n-6 | *cis*-8, *cis*-11, *cis*-14-eicosatrienoic acid | dihomo-γ-linolenic acid |
| 20:4n-6 | *cis*-5, *cis*-8, *cis*-11, *cis*-14-octadecatetraenoic acid | arachidonic acid |
| 23:0 | tricosanoic acid | tricosylic acid |
| 20:5n-3 | *cis*-5, *cis*-8, *cis*-11, *cis*-14-,*cis*-17-eicosapentaenoic acid | eicosapentanoic acid |
| 22:4n-6 | *cis*-7,*cis*-10, *cis*-13, *cis*-16-docosatetraenoic acid | Adrenic acid |
| 22:5n-6 | *cis*-4, *cis*-7, *cis*-10, *cis*-13, *cis*-16-docosapentaenoic acid | Osbond acid |
| 22:5n-3 | *cis*-7, *cis*-10, *cis*-13, *cis*-16, *cis*-19-docosapentaenoic acid | clupadonic acid |
| 22:6n-3 | *cis*-4, *cis*-7, *cis*-10, *cis*-13, *cis*-16, *cis*-19-docosahexaenoic acid | docosahexanoic acid |

**Table S3** – Total macronutrient intake of adult rats fed CONT, HVF and RUM diets.

|  | Experimental Groups^1^ | | | *P* value | F value |
| --- | --- | --- | --- | --- | --- |
|  | CONT | HVF | RUM |  |  |
| Carbohydrate intake (g | 123.78± 6.50^a^ | 59.11 ± 5.39^b^ | 51.63 ± 4.29^c^ | <0.001 | 519.86 |
| Protein intake (g) | 39.59± 2.15^a^ | 24.17 ± 2.23^b^ | 37.67 ± 3.20^a^ | <0.001 | 98.68 |
| Fat intake (g BW) | 10.95 ± 0.49^a^ | 33.82 ± 1.80^b^ | 35.20 ± 1.83^b^ | <0.001 | 391.54 |

1 -CONT: Control; HVF: hydrogenated vegetable fat; RUM: ruminant fat.

Values expressed as mean and standard error (One-way ANOVA, Tukey).

Averages on the same line with significant differences (*p*<0.05) are indicated by different letters.

**Table S4** – Weight of selected tissues of adult rats fed CONT, HVF and RUM diets.

|  | Experimental Groups^1^ | | | *P* value | F value |
| --- | --- | --- | --- | --- | --- |
|  | CONT | HVF | RUM |  |  |
| Tissue weight (g/100g BW) |  |  |  |  |  |
| Heart | 0.38±0.128 | 0.41±0.135 | 0.38±0.128 | 0.205 | 1.69 |
| Liver | 3.13±0.711 | 3.28±0.750 | 3.21±0.711 | 0.359 | 1.06 |
| Adipose tissue^2^ | 3.46^a^±3.91 | 1.94^b^±4.11 | 2.39^ab^±3.91 | 0.034 | 3.85 |

1 -CONT: Control; HVF: hydrogenated vegetable fat; RUM: ruminant fat.

2 – Abdominal and epididymal fat

Values expressed as mean and standard error (One-way ANOVA, Tukey).

Averages on the same line with significant differences (*p*<0.05) are indicated by different letters.
